# Non-AUG start codons responsible for *ABO* weak blood group alleles on initiation mutant backgrounds

**DOI:** 10.1038/srep41720

**Published:** 2017-01-31

**Authors:** Emili Cid, Miyako Yamamoto, Fumiichiro Yamamoto

**Affiliations:** 1 Laboratory of Immunohematology and Glycobiology, Josep Carreras Leukaemia Research Institute (IJC), Campus Can Ruti, Badalona, Barcelona, Spain; 2 Program of Predictive and Personalized Medicine of Cancer (PMPPC), Institut d’Investigació Germans Trias i Pujol (IGTP), Campus Can Ruti, Badalona, Barcelona, Spain

## Abstract

Histo-blood group *ABO* gene polymorphism is crucial in transfusion medicine. We studied the activity and subcellular distribution of *ABO* gene-encoded A glycosyltransferases with N-terminal truncation. We hypothesized that truncated enzymes starting at internal methionines drove the synthesis of oligosaccharide A antigen in those already described alleles that lack a proper translation initiation codon. Not only we tested the functionality of the mutant transferases by expressing them and assessing their capacity to drive the appearance of A antigen on the cell surface, but we also analyzed their subcellullar localization, which has not been described before. The results highlight the importance of the transmembrane domain because proteins deprived of it are not able to localize properly and deliver substantial amounts of antigen on the cell surface. Truncated proteins with their first amino acid well within the luminal domain are not properly localized and lose their enzymatic activity. Most importantly, we demonstrated that other codons than AUG might be used to start the protein synthesis rather than internal methionines in translation-initiation mutants, explaining the molecular mechanism by which transferases lacking a classical start codon are able to synthesize A/B antigens.

Histo-blood group ABO system consists of oligosaccharide A and B antigens and the antibodies against those antigens. Because these glycan antigens are expressed not only on red blood cells (RBCs), but also on epithelial and endothelial cells, matching the ABO blood groups is critical for safe blood transfusion and transplantation of cells, tissues and organs. Since its molecular elucidation in 1990^1^,^2^, numerous alleles at the human *ABO* genetic locus have been identified and characterized, and this locus has become one of the best-studied loci for genetic polymorphism and heterogeneity. The updated list of *ABO* gene alleles may be found in the Blood Group Antigen Gene Mutation Database (http://www.ncbi.nlm.nih.gov/gv/rbc/xslcgi.fcgi?cmd=bgmut/systems_info&system=abo)^3^. There are three major alleles: *A (A1*) alleles that encode *N*-acetyl-*d*-galactosaminyltransferases (histo-blood group A transferases), *B* alleles that encode *d*-galactosyltransferases (B transferases), and non-functional *O* alleles. Both the A and B transferases utilize the same acceptor substrate H (Fucα1-2Gal-), and transfer by the same α1,3-glycosidic linkage an *n*-acetyl-*d*-galactosamine (GalNAc) and a galactose (Gal) to synthesize oligosaccharides A and B antigens, GalNAcα1-3(Fucα1-2)Gal- and Galα1-3(Fucα1-2)Gal-, respectively. The O proteins possess neither activities, and H substance remains without further modifications.

In addition to those 3 alleles, there are additional alleles that specify weak expression of A or B antigens (phenotypically called as A_2_, A_3_, A_weak_, A_el_, B_3_, B_el_, etc.). The molecular mechanisms responsible include mutations in the promoter/enhancer regions of the *ABO* gene that cause a reduction in gene transcription, mutations in the splicing donor/acceptor sequences that cause inefficient splicing of correct messages, and mutations in the coding sequences resulting in structural deficiencies of the A/B transferases. In the last category, the majority of the mutations were identified in the catalytic domain of the coding sequences. Together with the determination of the three dimensional structures of the human A and B transferases^4^ and *in vitro* characterization of A and B transferases with mutations^1^,^5^, many of the non-synonymous mutations that were found present in the A/B subgroup alleles proved to be useful in characterizing the structure bases of specificity and activity of the A and B transferases. Although less frequent, *a priori* different group of weak alleles also exist. In this group, structural changes outside of the catalytic domain were identified. Those include initiation codon missense mutations (M1V in an *A*^*weak*^ allele, for instance) and nonsense mutations causing an early termination (E3X and R18X in *A*^*el*^ and *B*^*el*^ alleles)^6^,^7^,^8^.

We previously assessed the functionality of the *A* gene transcript starting from the alternative exon 1a^9^. The exon 1a does not contain an ATG codon, however, the transcript led to the expression of A antigens, suggesting that an internal codon was used for the initiation of translation. We also prepared a variety of expression constructs encoding N-terminally truncated A transferases with a deletion of the cytoplasmic tail and/or a portion/whole of the transmembrane (TM) domain, and analyzed their enzymatic activities. Several of the constructs exhibited the appearance of A antigens on the transfected human cells expressing cell-surface H substances although the abundance of A antigens was very small. Analyzing the effects of a translation-initiator mutation, a separate study by others showed that the mutation causes the A_weak_ phenotype^6^.

In spite of the fact that those studies delineated the significance of N-terminal domain(s) for the full activities of A and B transferases to produce large amounts of cell-surface A and B antigens, the subcellular localization of the proteins encoded by the respective constructs was not revealed. No clear answer was available explaining how some polymorphic alleles, without methionine 1 or a stop codon right after, were able to be translated. We re-visited the structure-function correlation of the N-terminally truncated A transferase proteins because intra-Golgi localization of the A and B transferases is vital for the biosynthesis of those oligosaccharide antigens. We examined the presence/absence of co-localization of those proteins with a Golgi marker protein derived from β1,4-galactosyltransferase 2 encoded by the *B4GALT2* gene and also with GOLGA2, a Golgi-resident protein. The results demonstrated that the N-terminal domain of A/B transferases might play a crucial role in the protein folding and in-Golgi localization of these clinically relevant transferases. Furthermore, during the course of investigation, we observed that a mutant A transferase construct having methionine to threonine substitutions at codons 1, 20, 26, 43, 53, and 69 induced the production of A antigens on transfected cells. Affinity purification and N-terminal sequencing of the protein demonstrated that the A transferase was translated using an unconventional non AUG-codon for initiation.

## Results

### Construction of A transferase N-terminal deletion mutants

**Figure 1:**
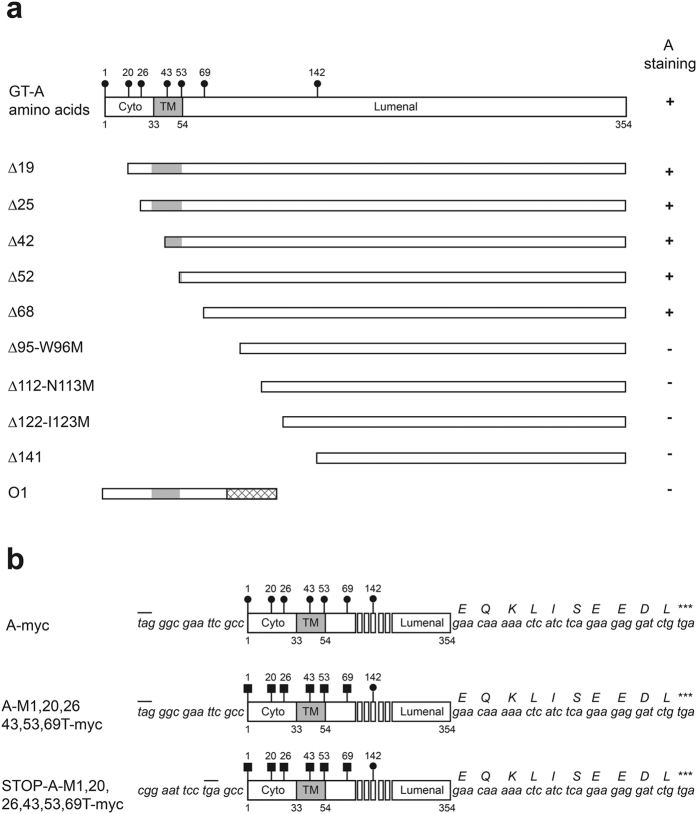
Schematic representation of the blood group A transferase coding sequence and protein. (**a**) Blood group A transferase is shown schematically. Numbers correspond to amino acid positions. Black circles are the methionines present in the N-terminal half of the enzyme. “Cyto” correspond to cytoplamic tail, and “TM” to transmembrane domain of the enzyme. The latter is also shown as a gray area. The “Lumenal” portion corresponds to the protein domain residing inside the Golgi. On the right column, “A staining” corresponds to the immunocytochemistry results. Plus sign (+) corresponds to a positive detection of A antigen and minus (−) a negative result. The different mutant construct protein products are depicted below. Δ19 corresponds to a deletion mutant starting at methionine 20 and therefore without amino acids 1 to 19. We named the following ones Δ25, Δ42, Δ52, Δ68 and Δ141 similarly. The Δ95-W96M lacks also 1–95 and starts in a newly created methionine at position 96 by introducing the missense mutation W96M. The same naming scheme was used for Δ112-N113M and 122-I123M. The O1 corresponds to the *O101* allele product. The crisscrossed area represents the additional amino acid stretch of frame-shifted reading frame after the nucleotide G261 deletion. (**b**) The myc-tagged A transferease and its threonine mutants are shown schematically. Solid squares represent introduced mutations, ATG → ACG exchanging methionine for threonine, in the indicated locations. The nucleotide sequence at 5′ of the first amino acid (M or T) is also shown. The small bars on top show in-frame STOP codons. At the C-terminal both the nucleotide and amino acid sequences corresponding to the myc-tag fragment are shown.

We first created a series of A transferase N-terminal deletion constructs starting at each internal methionine in order to explain the capacity of these methionines to act as start codons when their upstream initiation was impaired, due to missense mutations or early termination. The predicted topology of the protein is shown in Fig. 1: amino acids 1 to 32 correspond to the cytoplasmic tail, 33 to 53 to the transmembrane domain and the rest of the protein is located in the Golgi lumen as indicated by the annotation in public protein databases (http://www.uniprot.org/uniprot/P16442). All the methionines present within the N-terminal half of the protein are represented as filled circles and numbered according to their position.

Using an *A1* allele (*A101*) as template, the truncated open reading frames were PCR-amplified and cloned into pSG5. We also created their C-terminal myc-tagged versions to study their subcellular localization. A schematic representation of the resulting constructs made, Δ19, Δ25, Δ42, Δ52, Δ68 and Δ141, is also shown. The nomenclature is based on the amino acids deleted at the N-terminal of A transferase. For instance, Δ19 construct encodes for a protein lacking the amino acids 1–19 and starting at methionine at codon 20. We also constructed three more mutants starting within the lumenal portion of the protein, Δ95-W96M, Δ112-N113M and Δ122-I123M. These deletion mutants have a newly introduced methionine (M) start codon; each one starts right after eliminating secondary structure features of the globular domain as shown in the crystal structure^4^. They start after two β strands, β1 (82–84) and β2 (93–95) for Δ95-W96M, after α helix 1 (102–111) for Δ112-N113M, and after β3 strand (115–122) for Δ122-I123M. We also subcloned in the same vector the inactive allele *O1* which is identical to the *A101* allele except for the G261 nucleotide deletion.

### Preparation of FUT2 stably expressing cells and assessment of A antigen presence on cell membrane

In order to maximize the synthesis of A antigen by A transferases with non-optimal catalytic activity, we modified HeLa cells to overexpress α1,2-fucosyltransferase encoded by *FUT2* gene, and created HeLa FUT2 cells, following an strategy developed in the laboratory^10^. The expression of *FUT2* increased the availability of the H substance, the precursor to A and B antigens.

All the constructs shown in Fig. 1a were transiently transfected in those cells. After 72 h, the cells were fixed and subjected to immunocytochemistry against A antigen. Positive staining only appeared on cells transfected with A transferase construct, the truncated mutants Δ19, Δ25, Δ42, Δ52, and Δ68 as marked by positive signs in Fig. 1. On the contrary, Δ95-W96M, Δ112-N113M, Δ122-I123M, Δ141 and O1 were all negative (see Supplementary Figure SF1 for representative images of stained cells). The ABC protocol for immunocytochemistry used consists of a streptavidin-biotin complex conjugated to an enzyme producing the chromogenic reaction. The amplifying nature of the staining procedure prompted us to examine another method to assess the capacity of each mutant to produce A antigen on the cell surface in a more quantitative manner. We also wanted to assay this capacity in a shorter time window in order to avoid product accumulation, which would not reflect enzymatic capacity adequately.

### Measurement of A antigen production by cytometry

We subcloned the deletion mutants that showed positive staining into a retroviral vector containing an IRES (Internal Ribosome Entry Site) sequence followed by GFP (Green Fluorescent Protein). HeLa FUT2 cells were transiently transfected with these retroviral constructs as well as Δ141, which showed no activity. Only those cells exhibiting green fluorescence were expressing the A transferase constructs. Around 32 h post transfection, cells were detached with 5 mM EDTA and washed extensively with PBS and stained with monoclonal anti-A antigen antibody followed by AlexaFluor 647-conjugated anti-mouse IgM secondary antibody and analyzed by cytometry (36 h post-transfection).

Two different controls to test the secondary antibody background and the specificity of anti-A staining were used: cells transfected with an empty vector expressing only GFP not stained with any primary antibody and cells expressing A transferase treated with anti-B antibody as a suitable isotype antibody control. A third control was added and included in all the analysis: cells transfected with the empty vector, therefore expressing only GFP and treated with anti-A and secondary antibodies, which accounts for the background fluorescence of the primary antibody used. The same detection settings were used for all the following experiments. Forward and side scatter values were used to select the viable single cell population and the fluorescence at 550 nm corresponding to GFP to select those cells expressing the transgenes.

**Figure 2:**
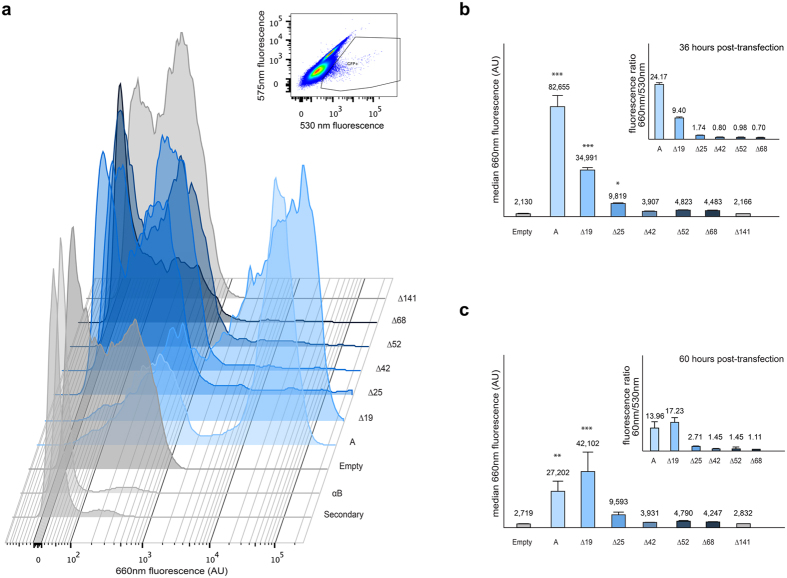
Cytometry analysis of A antigen synthesis capacity. (**a**) HeLa FUT2 cells transfected with A transferase constructs were selected by increased 530 nm fluorescence from the global population as seen in the upper inset. The composite histogram shows the 660 nm fluorescence of those cells corresponding to the presence of A antigen by detection of a secondary antibody conjugated to Alexa647 binding in turn to an anti-A antigen antibody. The “Secondary” histogram corresponds to A transferase transfected cells treated without primary antibody, the “αB” to the same cells treated with anti-B antibody instead of anti-A antibody. The rest of cells were stained both with anti-A and the secondary antibody. The “Empty” graph shows the staining of cells transfected with an empty vector, “A” with the A transferase construct, and further on. The X-axis shows fluorescence at 660 nm on an exponential scale. The height of the graph corresponds to the number of cells for each fluorescence value. (**b**) The median of 660 nm fluorescence is shown of each construct from five independent experiments after 36 h post-transfection. ***P < 0.001, *P > 0.05 assigned by the Duncan test. The inset graph shows the mean of the 660 nm/530 nm fluorescence for each data point. (**c**) The median of 660 nm fluorescence after 60 h post transfection. ***P < 0.001, **P > 0.01.

Figure 2a is a superimposition of histograms corresponding to the fluorescence at 660 nm for all the samples analyzed. The anti-B antibody and secondary antibody only controls showed no fluorescence. Cells transfected with empty vector presented a level of 660 nm fluorescence similar to the inactive Δ141 sample. In contrast, A construct is highly positive, as is Δ19; after that the fluorescence decreases gradually for the remaining mutant constructs.

For each experiment, we measured the median red fluorescence at 660 nm of A antigen-positive cells, defined as those above the background level, for each construct. The means of the medians are shown in Fig. 2b. As expected, non-truncated A transferase gave the highest values. Regarding the truncated mutants, Δ19 and Δ24, while still conserving the transmembrane domain, showed diminished but clear capacity to produce A antigen when transfected into HeLa FUT2 cells (42% and 12% of the A median fluorescence, respectively). Once the truncation spanned most, or all, of the TM domain as of the truncated mutants, Δ42, Δ52 and Δ68, the capacity to produce surface A antigen drastically diminished. However, their enzymatic activity is not null. Although no statistically significant differences were found between those constructs and the empty construct control, they have higher median values than the background. Furthermore, clearly positive A staining was observed with those constructs by immunohistochemistry where signal is amplified (Supplementary Figure SF1). Finally Δ141, with a truncation well within the lumenal portion, and hence in the catalytic portion of the enzyme, showed no antigen production on the membrane and its fluorescence median is practically identical to the empty control. This is a real null construct, and no A antigen expression was observed even by the more sensitive immunohistochemical method.

We repeated the fluorescent measurements at 60 h post-transfection. Results were similar, but cells transfected with original A transferase seem to have lost some fluorescence intensity. This may reflect protein turnover or cell death due to overexpression of the enzyme or its product. The rest of constructs show a very similar behaviour as in the 36 h experiment. Again only those constructs showing substantial activity (A, Δ19) are statistically different from the empty control although the others (except for Δ141) consistently showed higher median values than the background.

Inset graphs in Fig. 2b and c show the median for the 660 nm/530 nm fluorescence ratio. It is a measure of the “efficiency” of the protein to produce antigen. The expression of GFP (measured by the fluorescence emission at 530 nm), is concordant with the expression of the construct as it is the result of the IRES sequence on the same transcript, and therefore, it is an indication of the level at which the A transferase is transcribed. For a given amount of transcribed protein, cells transfected with full A transferase construct exhibited 24 times more fluorescence intensity at 660 nm than with Δ25, while Δ19 had an intermediate value of 9.4 at 36 h post-transfection. At longer time of 60 h, this “efficiency” becomes similar between A and Δ19 (14 vs. 17) due to the accumulation of A antigen.

### Subcellular localization of N-terminal truncated A transferases is crucial to their antigen synthesis ability

**Figure 3:**
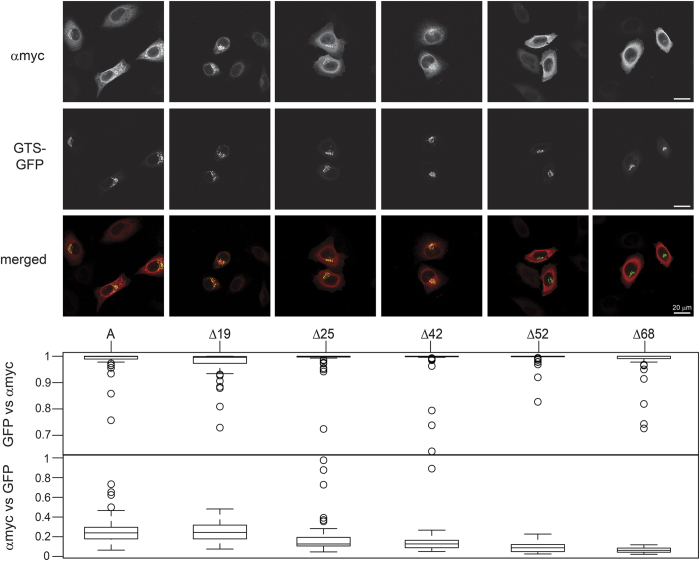
Subcellular localization of overexpressed active A transferase internal methionine deletion mutants. HeLa FUT2 cells were transfected with A transferase mutants able to produce A antigen as detected by immunocytochemistry. Lipofectamine 2000 was used. A Golgi-targeted GFP, GTS-GFP, was co-transfected. Single optical slice images were taken using confocal microscopy. Anti-myc (αmyc in the top panel) shows the signal from myc-tagged proteins detected with a primary anti-myc antibody followed by secondary Alexa568-anti mouse IgG antibody. The merged images are colour coded red for myc-tagged proteins and green for GTS-GFP, showing colocalization in yellow. Zeiss ZEN software was used to determine the colocalization factor values between the αmyc signal detecting the protein and GTS-GFP marking the Golgi apparatus for each image. More than 50 images were analyzed from four independent experiments. The values obtained were plotted using R software as boxplots.

We also wanted to determine the intracellular localization of the mutant proteins to correlate their activity with their presence in the Golgi apparatus. First we created a Golgi-targeted GFP (GTS-GFP) marker by fusing the first 60 amino acids of the *B4GALT2* encoded β1,4-galactosyltransferase 2 to the N-terminus of GFP. We subcloned this open reading frame in the pRK5 vector. The transient expression in HeLa FUT2 cells showed perinuclear distribution typical of the Golgi apparatus as seen in Fig. 3 (GTS-GFP panels).

**Figure 4:**
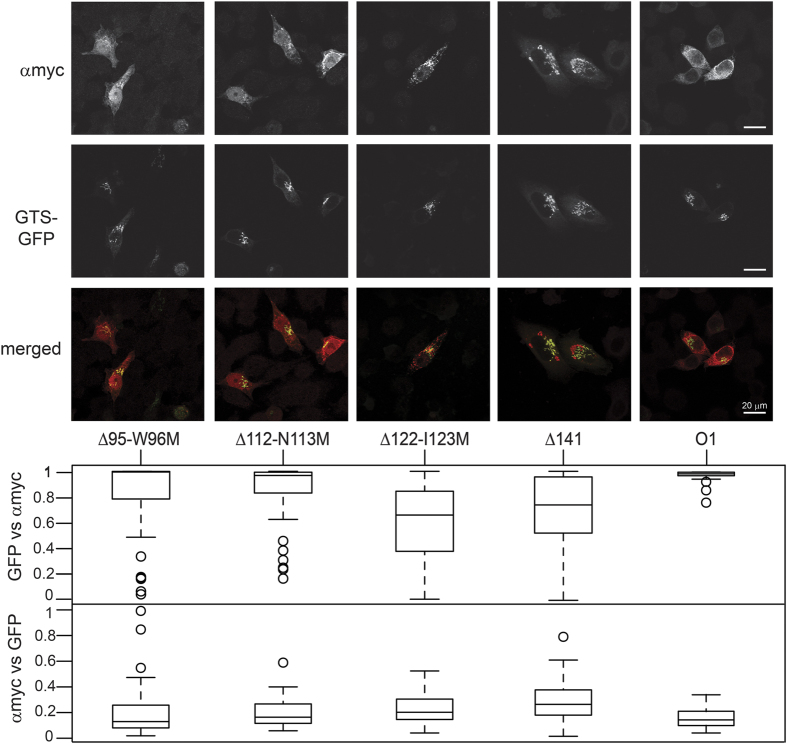
Subcellular localization of overexpressed inactive A transferase internal methionine deletion mutants. HeLa FUT2 cells were transfected with A transferase mutants that are unable to produce A antigen as detected by immunocytochemistry. The same experimental conditions described above in the legend of Fig. 3 were used for DNA transfection and fluorescence detection.

The localization within the cell of the myc-tagged N-terminally truncated proteins present great differences as it is shown in Figs 3 and 4. Non-truncated myc-tagged A transferase exhibits a clear colocalization with the Golgi marker; the overlap can be appreciated in yellow in the merged images. It also shows signal in other cellular structures reflecting the dynamic distribution of the enzyme. The Golgi localization of the myc-tagged proteins was quantified using confocal microscopy by measuring their degree of colocalization with GTS-GFP. The median of the colocalization coefficient of anti-myc (αmyc) signal with GTS-GFP signal for each construct is shown as a boxplot in Fig. 3. The colocalization is the same for the whole enzyme (0.24) and Δ19 (0.24). On the other hand, Δ25, although with a higher variability, already shows a lowered Golgi localization, similar to Δ42 (0.13 both). In the case of Δ42, its transmembrane domain has been truncated (only about half of it remains: the last 12 amino acids out of 22) but it is still able to localize, albeit partially, in its intended compartment. Regarding Δ52 and Δ68, the former has only two amino acids of the transmembrane region, which is clearly not sufficient for targeting the protein to the Golgi and, as Δ68, does not colocalize at all with GTS-GFP (0.09 and 0.06 respectively), clearly being illustrated in the merged picture where no yellow signal is present. Moreover, Δ52 and Δ68 show a more homogenous distribution throughout the cell, a pattern compatible with a typical cytoplasmic soluble protein. The complementary data, that is, the colocalization coefficient of GTS-GFP vs. αmyc signal is constant for all samples and very close to 1. This indicates that there is always red signal together with green, or in other words, there is always overlapping.

Figure 4 shows the subcellular localization of the inactive N-terminally truncated proteins together with the Golgi marker GTS-GFP. Δ141, the last initiation methionine mutant, and in contrast with all the others, is completely inactive. As it starts well within the lumenal portion of the protein, it has lost important structural features of the catalytic domain. Furthermore, the tagged protein signal showed bigger aggregation. This aberrant localization is accompanied by an increased overlap with GTS-GFP, contrasting with the soluble localization of Δ52 and Δ68 myc-tagged proteins (0.26). We have also evaluated the subcellular distribution of Δ95-W96M, Δ112-N113M and Δ122-I123M mutants that, even though they do not represent transcripts initiated by any internal methionine, they would add more information about the dependence of the protein distribution on the N-terminal domain length. The first two, Δ95-W96M and Δ112-N113M, showed an apparent nuclear accumulation. The nuclear distribution was accompanied by some cytoplasmic aggregation of the fluorescent signal and an intermediate degree of colocalization with GTS-GFP (0.13 and 0.16). The last mutant Δ122-I123M behaved similarly as Δ141 and showed enhanced colocalization with GTS-GFP (0.20). The upper panel shows the colocalization coefficient of GFP-GTS vs. αmyc signal. It is also close to 1 for Δ95-W96M and Δ112-N113M, as in the case of active mutants, while Δ122-I123M and Δ141 mutants had reduced colocalization (0.66 and 0.74 respectively). The last two also showed increased aggregation of the myc signal.

**Figure 5:**
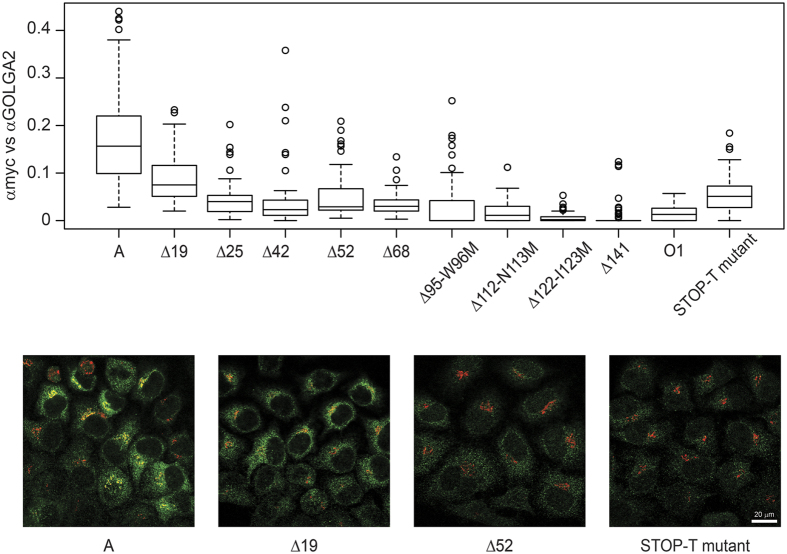
Subcellular localization of stably transfected A transferase mutants. HeLa FUT2 cells were stably transfected with A transferase mutants by retroviral infection and FACS selection. Single optical slice images were taken using confocal microscopy. Anti-myc shows the signal from myc-tagged proteins detected with a primary anti-myc antibody followed by secondary Alexa568-anti mouse IgG antibody. Golgi protein GOLGA2 was detected using a rabbit anti-GOLGA2 antibody followed by secondary Alexa647-anti rabbit IgG antibody. The merged images are colour coded green for myc-tagged proteins and red for GTS-GFP, showing colocalization in yellow. Zeiss ZEN software was used to determine the colocalization factor values of the anti-myc signal detecting the tagged protein and GOLGA2 marking the Golgi apparatus. Representative images of four of the constructs are shown. At least 50 cells were analyzed per construct. The values obtained were plotted using R software as boxplots.

As these experiments were performed using a CMV promoter and transient transfection conditions, we were concerned that some of these subcellular patterns, especially the aggregation, were due to a high level of overexpression. Therefore, we also stably transduced HeLa FUT2 cells with these myc-tagged mutants in a viral vector and analyzed their subcellular localization avoiding such overexpression. The results are shown in Fig. 5. The Golgi apparatus was detected using an antibody against the resident protein GOLGA2. All myc-tagged proteins presented a speckled pattern when detected using anti-myc antibody. The colocalization quantification shown in the upper panel also showed that the overlapping between the myc and the Golgi signals decreased as the N-terminal deletion increased. The weighted colocalization median of αmyc against GOLGA2 for each construct are as follows: 0.156 (A), 0.075 (Δ19), 0,04(Δ25), 0.023(Δ42), 0.029 (Δ52), 0.03 (Δ68), 0.00 (Δ 95-W96M, Δ122-I123M and Δ141), 0.01 (Δ112-N113M). The images show greater expression and a clear colocalization (in yellow) of A-myc and Δ19-myc with the Golgi marker, while all the others present lower or no colocalization at all. Furthermore, the expression of these other mutants appears to be lower. The STOP-T-mutant quantification indicates some colocalization but we believe that the decreased expression of these proteins makes their detection and therefore their colocalization measurements more difficult. Images for the myc-tagged proteins A, Δ19, Δ52 and STOP-T-mutant are shown at the bottom of Fig. 5. A representative image for each mutant can be found in Supplementary Figure SF2. The O1 mutant showed reduced Golgi localization (0.14 in Fig. 4 and 0.013 in Fig. 5) when compared to A transferase.

### A mutant lacking Met 1 start codon is translated using a non-AUG codon

**Figure 6:**
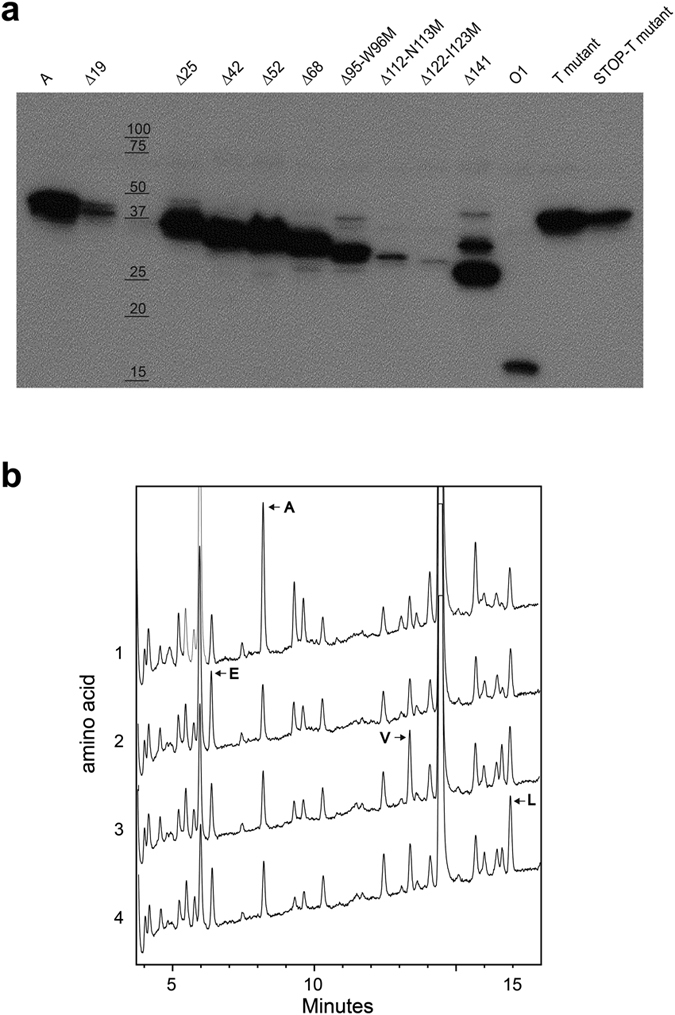
Determination of the translation start site of the threonine substitution mutant. (**a**) HeLa FUT2 cells were transfected with the myc-tagged versions of A transferase (A, Δ19, Δ25, etc.), A-M1,20,26,43,53,69T-myc mutant (T mutant), and the same construct with an in-frame STOP codon at 5′ of the first threonine (A-STOP-M1,20,26,43,53,69T-myc denoted as STOP-T mutant in the figure). Cell extracts were subjected to SDS-PAGE (10% polyacrylamide) and blotted onto PVDF membrane. Signals were obtained using a monoclonal anti-myc antibody followed by horseradish peroxidase-linked anti-mouse IgG antibody incubation and enhanced chemiluminescence reagent. Signals were detected on chemiluminescence sensitive film. The numbers indicate the sizes and positions of molecular weight markers in kDa. (**b**) The N-terminal sequence chromatograms obtained by the Procise 494 analyzer are shown for A-STOP-M1,20,26,43,53,69T-myc. In each cycle the major amino acid detected has been marked by an arrowhead and its one-letter symbol.

Next, we wanted to compare the capacity of the truncated enzymes with the product of the A-M1,20,26,43,53,69 T mutant, which possesses the methionine to threonine substitutions at codons 1, 20, 26, 43, 53, and 69. The latter was reported to produce A antigen in transfected cells^6^ and we corroborated it by transfection and immunocytochemistry by DAB staining. By cytometry it showed a level of median fluorescence close to the unmodified A allele or Δ19 (a 58% of A median fluorescence). In order to exclude the possibility of an upstream 5′ start codon we introduced a closer in-frame STOP codon right before the threonine at position 1. The resulting construct was also positive for A staining. These proteins are schematically represented in Fig. 1b. We decided to investigate the molecular weight of the protein responsible for the A antigen appearance. The Western blot against myc (from now on myc) detecting the myc-tagged versions of the N-terminal deletion enzymes, including A-M1,20,26,43,53,69 T and STOP-A-M1,20,26,43,53,69 T, is shown in Fig. 6a. The deletion mutants have smaller sizes than A transferase as expected. On the other hand, the proteins produced by the two threonine mutants described above are identical in size and indistinguishable from the unmodified enzyme.

In order to know which initiation codon was used to translate a protein of this size in the absence of any methionine codon at the N-terminal region, we turned to protein N-terminal sequencing. To do that, HeLa FUT2 cells were infected with retroviral vectors encoding the myc-tagged versions of A and A-STOP-M1,20,26,43,53,69 T mutant followed by an IRES sequence and GFP. Stable transformants were selected and seeded as single cells in 96 well plates. Individual clones were grown and tested by immunoblotting for high expression of the proteins. The clones with highest myc/tubulin ratio were selected and expanded. Cellular extracts were subjected to affinity chromatography with agarose coupled to anti-myc monoclonal antibody and eluted with the myc peptide. The purified proteins were then blotted onto PVDF membrane and stained with Ponceau S. The band corresponding to the M1,20,26,43,53,69 T mutant and control A transferase were excised and sequenced by an automated protein sequencer. The results, plotted in Fig. 6b, indicated that the mutant protein started with alanine-glutamic acid-valine-leucine (AEVL), which correspond to amino acids 2 to 5 of the A transferase protein, exactly the same as the unmodified A protein (see Supplementary Data SD1 for the complete N-terminal protein sequencing results). Therefore, the translation machinery was able to use the ACG triplet at Thr 1 to start translation from the same location as the wild type enzyme.

## Discussion

We were interested in finding out a possible molecular mechanism explaining the presence, albeit reduced, of A or B antigen in *ABO* alleles with the translation on initial methionine codon disrupted by missense mutations or by early termination codons in the 5′ end of the coding sequence. Different alleles of these kind have been described: *Aw13* (2 T > C) M1T with initiator methionine codon disrupted specifying an A_w_ phenotype^6^ or *A309* (1 A > G) M1V producing an A_3_ phenotype^8^. The same authors described the early termination mutations R18X and E3X (on *B* allele) that were associated with the weak phenotypes A_el_ and B_el_, respectively, and hypothesized that N-terminally truncated enzymes translated from remaining internal methionines were responsible for the antigen presence.

We tested this hypothesis and analyzed if proteins starting at internal methionines were able to synthesize the antigen. This had been investigated before as the catalytic activity of these truncated proteins was measured in homogenates^9^. However, in the work described here we also studied the subcellular localization of these deletion mutants. The first truncated protein Δ19 and full-length A transferase were similarly located within the Golgi apparatus but Δ19 produced less A antigen on cell surface. We are not sure if that is the result of a decreased activity although its “efficiency” ratio might indicate that. Δ25 still contains a complete transmembrane domain and exhibits significant activity and an intermediate level of Golgi localization. These results further stress the fact that a complete TM and lumenal domains are necessary for a substantial A antigen presence on the membrane. In previous reports^6^,^9^, the transmembrane domain was assumed to be situated between amino acids 17 and 37. Therefore, Δ25 was considered to be a mutant in the “stem region” while more recently the TM region is being localized at 33–53, leaving Δ25 as a Golgi-located mutant as our results corroborate. Δ42 seems to be a special case, conserving only about half of the TM domain. It is still localized in Golgi, at a similar level as Δ25, but in contrast, it is not able to deliver a significant amount of A antigen indicating both the crucial role of exon 3 (which codifies for the 90% of the TM domain) and the fact that interfering with subcellular localization signals is determinant for altered glycosyltransferase activity. The importance of exon 3 in antigen production can be further emphasized by other reports. For example, Cai *et al*.^8^ found that the conservative R40K missense mutation in the TM region results in an A_x_ phenotype (mainly characterized by minimal amount of A antigen in saliva and detected by hemagglutination with anti-A,B but not with anti-A antibodies).

Hata *et al*.^9^ examined A transferase activity for Δ52 and Δ68 constructs in an *in vitro* enzymatic assay utilizing transfected cell extracts, ^14^C-radiolabeled UDP-GalNAc, and 2′-fucosyllactose. Both constructs showed specific activities in the range of the wild type enzyme demonstrating that the catalytic activity was not affected. The fact that this work was not able to detect surface antigen by cytometry in a statistically significant amount could be the result of its incorrect localization as shown by our microscopy experiments. Even though they conserve enzymatic capacity, they do not biosynthesize a considerable amount of antigen, as they require access to substrates at appropriate concentrations. The lack of proper pools of UDP-GalNAc and H substance in the cytosol and improper localization of the protein greatly limit the ability of this enzymes to transfer GalNAc to H substance. However, a very minor fraction of the protein may enter the Golgi apparatus and we believe that *this* fraction may be the one responsible for the small amount of antigen present on the cell surface.

Δ141 is completely inactive. No specific activity for this protein was found by Hata and colegues^9^, coinciding with our results. When overexpressed, the aberrant distribution of this truncated protein has an effect on the GTS-GFP reporter localization as these two proteins aggregate and colocalize in our model, while none of the other N-terminal deletion mutants produce these phenomena. In order to understand this, we analyzed truncated mutants with intermediate lengths between Δ68 and Δ141. We also performed an alternative detection of myc-tagged mutants using a stable transfection system characterized by lower levels of transgene expression. We decided to position the starting methionines right after secondary structure features. Although the localization observed for mutants Δ95-W96M and Δ112-N113M in the transient overexpression experiments is mostly nuclear and their low masses (approximately 28 and 26 kDa) are well below the exclusion limit for nuclear pore entry by diffusion (60 kDa)^11^, we did not observe that accumulation when cells were stably transfected (Supplementary Figure SF2). Neither aggregation nor high colocalization with GTS-GFP or GOLGA2 occurred. On the other hand, the distribution of Δ122-I123M is quite similar to Δ141 in the transient overexpression experiments. When the stable transfectants are analyzed, the colocalization coefficient with GOLGA2 of all four mutants is 0 or close to 0, clearly showing that they are not able to locate properly. Together with the immunocytochemistry experiments (Supplementary Figure SF1), these results suggest that they have no activity at all. In conclusion, any type of mutation causing a truncation past the TM domain spoils cell surface antigen expression, and those N-terminal deletions reaching past codon 95 will render the encoded protein catalytically inactive.

Seltsam *et al*.^6^ showed that the allele *Aw13* (2 T > C) with initiator Met codon exchanged by threonine produced a weak A phenotype and hypothesized that remaining internal methionines would be the starting amino acids of N-terminally truncated enzymes which in turn would be responsible for the antigen presence. In order to demonstrate that hypothesis, they followed a similar approach as ours but in that case they mutated sequentially and systematically all the ATG methionine codons to ACG encoding threonine. The results indicated that all the mutants, from M1T to M1,20,26,43,53,69 T had exactly the same capacity to produce A antigen on cell surfaces as measured by cytometry. In the light of our results, we deduced that they were essentially measuring the same protein, that is, an A transferase translated from the first mutated ACG codon and having the same length as the wild type enzyme. In this case, the additional and internal methionine to threonine mutations have apparently no deleterious effects. We may conclude that internal methionines are not necessary to initiate the transcription, but a codon similar to AUG that is close to the translational start, is. This phenomenon has been described in mammalian cells where certain codons differing from AUG by only one nucleotide may function as start codons^12^. The ribosome scans 5′ to 3′ from the beginning of the transcript and may choose another codon similar to AUG if the context is appropriate^13^. In fact, initiation at non-AUG codons is emerging as a new mechanism of protein expression regulation. Ingolia *et* *al*.^14^ characterized the abundance, type and identity of alternate open reading frames in mouse embryonic stem cells and have shown that a great number of alternate initiation sites are non-AUG. The same explanation could be extended to the mutant allele *A309* (1 A > G) M1V as described above^8^. In the cases of R18X and E3X (on *B* allele) other AUG-like codons further down the premature stop would be responsible for the translation. Indeed, the A_el_/B_el_ phenotypes present fewer antigens than the A_3_ phenotype. This fact may respond to a longer distance of the new start codon from the original start or a less favourable sequence milieu. In summary, in the human context, once the canonical start codon is eliminated or the protein is early terminated, alternative mechanisms might act in order to allow the expression of an *ABO* encoded enzyme.

*In vivo*, inefficient translation of these mutants is probably responsible for the reduced expression of these proteins. Although their intrinsic activity is probably very similar to the wild-type enzyme, the amount of protein synthesized might be greatly diminished. In the case of the *Aw301* allele corresponding to the M1T mutant, the efficiency of translation from a non-AUG start codon (ACG as we have demonstrated) produces the A weak phenotype which is characterized by a residual amount of A antigen present on cell surfaces. We tested this using two different promoters in dissimilar genomic contexts, a cytomegalovirus promoter in an episomal plasmid and a retroviral long terminal repeat promoter in a random genomic integration site. We obtained higher translation rates, allowing us to describe this phenomenon.

## Methods

All the DNA constructs used in this work were prepared using standard molecular biology protocols throughout. After PCR amplification and cloning, Sanger sequencing was performed to check that no spurious mutations were introduced. Oligonucleotide sequence information is available in Supplementary Methods SM1.

### Glycosyltransferase genes cloning

For the HeLa FUT2 creation, total RNA from human cell lines (MCF-7, MDA-MB468, MDA-MB231, BT-20 and T-47D from ATCC; #HTB-22, HTB-132, HTB-26, HTB-19, and HTB-133 respectively) and normal human breast epithelial cells (Cambrex) was obtained by Trizol (Life Technologies) extraction following manufacturer’s instructions, and pooled. Reverse transcription was performed with 1 μg of total RNA using Superscript III (Invitrogen) and random primers. The resultant cDNA was used to clone *B4GALT2* gene cDNA with oligonucleotide primers EC19/20 by PCR. *FUT2* gene cDNA was amplified by nested PCR using the cDNA as template and oligo primers TF49(F)/TF48(R) in the first PCR and TF49(F)/TF50(NESTED R) in the second PCR. The open reading frame of *B4GALT2* was subcloned into pcDNA3.1 and *FUT2* into pMigR1b^10^.

### Blood group A transferase constructs

The coding sequence of A transferase or O1 protein cloned in pSG5 plasmid (Stratagene) was available in the laboratory as templates. Deletion constructs were obtained by PCR using Pfx SuperMix and custom synthesized oligo primers. The names and nucleotide sequences of primers are found in Supplementary Methods SM1. The coding sequences were cloned using *EcoRI*/*BamHI* restriction sites into pSG5 vector. Their myc-tagged versions were constructed amplifying each construct using the T7 primer and EC84 reverse primer followed by *EcoRI* and *BamHI* digestion and subcloning them into pSG5 vector.

The methionine mutants were prepared through a two-round PCR amplification using specific primers with nucleotide substitutions. In brief, the N-ter and C-ter DNA fragments containing nucleotide substitutions at one end were separately amplified in the initial round of PCR. Then those two PCR reaction products overlapping at the mutation site were mixed, and a secondary round of PCR was performed to amplify full-length DNA fragments.

The constructs needed for cytometry analysis were constructed by PCR amplification using T7 and primer EC110 and the truncated A transferases and threonine mutants in pSG5 plasmids as templates. After digesting with *EcoRI*, the fragments were subcloned into the same site of pMigR1g. Orientation of the inserts was checked by restriction analysis.

For the stably transfected cells used in the subcellular localization experiments, we modified the pMigR1g retroviral vector. We introduced a *BamHI* site using an oligo containing it at 3′ of the *EcoRI* site (oligonucleotide IM927) that was used to amplify the portion of the vector between the *EcoRI* and *NcoI* sites (with oligonucleotide IM928). Then the amplicon cut with these enzymes was used to replace the corresponding fragment of pMigR1g vector resulting in the pMigR2g vector. *EcoRI* and *BamHI* sites were used to directly sublcone the myc-tagged constructs from pSG5 vector into the new pMigR2g.

### GTS-GFP

Golgi Targeting Sequence–GFP (GTS-GFP) was created by amplifying GFP cDNA sequence by PCR using oligonucleotides EC111/EC112 and pMigR1 as template. The resulting fragment was cut with *NheI*/*XhoI* and subcloned into the pcDNA3.1-B4GALT2 plasmid also cut with the same restriction enzymes. The resulting plasmid codifies for the first 60 amino acids of β1,4-galactosyltransferase 2, known to direct the protein to the Golgi apparatus, followed in frame by the GFP sequence. The GTS-GFP was subcloned into the *XhoI* site of pRK5 vector by PCR amplification using oligos EC99/EC112 and restriction. Appropriate clones that contained the coding sequence in the correct orientation were selected.

### DNA purification

Plasmids were amplified in TOP10 cells and Midiprep DNA was performed using Life Technologies PureLink Filter Midiprep Kit.

### Cell stable transduction and culture

HeLa cells (ATCC #CCL-2) were transduced with human FUT2-IRES-mTagBFP retrovirus followed by FACS isolation as described in Cid *et al*.^10^ and named HeLa FUT2. Similarly, HeLa FUT2 cells were transduced stably with A myc-tagged mutants subcloned in pMigR2g for the subcellular localization experiments detailed in Fig. 5. For the N-terminal amino acid sequence determination MigR1-A and pMigR1-STOP-A-M1,20,26,43,53,69 T were transduced following the same strategy. Cells were maintained in a 5% CO_2_ incubator at 37 °C in Dulbecco’s Modified Eagle’s Medium (DMEM) with 10% fetal bovine serum (FBS) and streptomycin/penicillin.

### DNA transfection

For overexpression subcellular localization experiments, myc-tagged N-terminal truncated A transferase constructs were transfected, together with GTS-GFP, in HeLa FUT2 cells plated in 8-well chambers (EZ slide Millipore) using Lipofectamine 2000 reagent in OPTI-MEM medium following the protocol provided by the manufacturer (Life Technologies). Transfection medium was replaced after 15 h with DMEM + 10% FBS and 24 h after transfection the cells were fixed with 4% paraformaldehyde, and washed extensively with PBS.

For cytometry analysis HeLa FUT2 cells were seeded in 35 mm culture dishes (6.5 × 10^5^ cells per dish) the day before transfection. Following the manufacturer’s protocol Lipofectamine 2000 and OPTI-MEM medium were used for transfection. 12 h later the medium was replaced with DMEM + 10% FBS and were maintained in a CO_2_ incubator for one or two more days. Before immunostaining cells were washed with PBS and detached from the plate using 5 mM EDTA in PBS.

### Immunostaining

For subcellular localization experiments, cells grown on glass coverslips precoated with 100 μg/ml poly-*d*-lysine were permeabilized for 10 min with PBS plus 0.2% Triton X-100 and blocked using PBS plus 3% Bovine Serum Albumin (BSA) and 0.2% Triton X-100 for 20 min. In the case of cells transiently overexpressing myc-tagged proteins, immunostaining was performed using a mouse monoclonal antibody against the myc epitope (9E10 from Merck Millipore) and AlexaFluor-568 anti-mouse IgG antibody (Life Technologies), both diluted in PBS with 1% BSA. The coverslips were mounted with ProLong Antifade Gold (Life Technologies). For cells stably expressing the myc-tagged proteins the same α-myc antibody was combined with a rabbit anti-GOLGA2 (Elabscience), and the secondary detection was performed using AlexaFluor-568 anti-mouse IgG and AlexaFluor-647 anti rabbit IgG (Life Technologies).

Cells destined to cytometry analysis were maintained at 4 °C during the whole procedure. First they were centrifuged at 500× *g* for 5 min and EDTA-PBS was removed. Their unspecific binding sites were blocked with PBS plus 1% BSA for 30 min, primary antibody (anti-A and anti-B mouse IgM from Ortho Clinical Diagnostics at 5-fold dilution) was added in the same buffer and incubated for 1 h, followed by two washes with PBS with 1% BSA using the same centrifugation settings, then by incubation with the secondary antibody (AlexaFluor-647 anti-mouse IgM from Life Technologies) followed by two washes. Cells were resuspended in PBS prior to analysis.

### SDS-polyacrylamide gel electrophoresis and immunoblotting

Standard protocols were used for SDS-polyacrylamide gel electrophoresis (SDS-PAGE) and immunoblotting using acrylamide/bis-acrylamide 29:1 and Immobilon-P membrane (Millipore). Western blot was performed using TBS with 1% Triton X-100 for washes, blocked with TBS plus 1% Triton X-100 and 3% Non-fat dry milk and incubated with anti-myc 9E10 clone at 1/1000 in the same solution followed by washes and incubation with anti-mouse IgG-HRP antibody at 1/1000 (Dako). After 5 washes, the membrane was incubated with ECL substrate and photosensitive film was exposed to the emitted chemiluminescence. Film was developed using an automated FUJI Medical Film FPM100A processor.

### Cytometry

For sorting (FUT2 and myc-tagged A and STOP-A-M1,20,26,43,53,69 T), cells were resuspended in DMEM supplemented with 2% FBS and streptomycin/penicillin and sorted using a FACS Aria II sorter (BD Bioscience). For the detection of A antigen on cell surface, cells were resuspended in PBS, and a FortessaLSR cytometer (BD Bioscience) was used for fluorescence detection. Results were recorded and analyzed with BD FACSDiva software and plotted using FlowJo.

### Microscopy and colocalization quantification

Confocal microscopy was used to determine the subcellular localization of myc-tagged constructs and their degree of colocalization with GTS-GFP or GOLGA2. Only one optical plane image was obtained per field and colocalization analysis was performed using the ZEN2 software and the weighted colocalization coefficient for the myc signal channel versus the Golgi localized GFP/GOLGA2 channel logged. Confocal images were obtained using a LSM710 AxioObserver Zeiss microscope and a Zeiss Plan-Apochromat 63x/1.40 oil DIC M27 objective. At least 50 cells were analyzed for each construct.

### Statistical analysis

Median fluorescence calculation and significance analysis (Duncan test) were performed using the R statistical program (version 3.1.0). Each construct was tested at least 4 times.

For the colocalization analysis, the data obtained by the Zen2 software was also introduced in the R program and boxplots and median calculations obtained. At least 50 cells for each construct were analyzed.

### Affinity purification and N-terminal sequencing

Cells were lysed using TBS with 1% Triton X-100 plus Mini protease inhibitor cocktail (Roche) and 1 mM PMSF. After keeping the extract on ice for 15 min it was sonicated using an Omni-Ruptor 4000 sonicator at output 3, for 1 min at 50% intermittence (OMNI International). The extract was then centrifuged at 15,000× *g* speed in a Sorvall RC6 + centrifuge and the pellet discarded. A Bio-Rad Poly-Prep chromatography column was packed with anti-myc agarose resin (Thermo Scientific) and prewashed with TBS. The clarified extract was recycled through the resin multiple times. The resin was washed with 20 volumes of TBS+1% Triton X-100 and with 20 more volumes of TBS and eluted with 1 mg/ml myc peptide solution in TBS (Biotool.com). The eluted fraction was concentrated using an Amicon centrifugal device. After gel electrophoresis and blotting, the membrane was stained with Ponceau S and dried. The band corresponding to the selected protein was excised using a scalpel and sent to the Protein Chemistry Service of the Centro de Investigaciones Biológicas of Consejo Superior de Investigaciones Científicas, Madrid, Spain, where N-terminal sequence determination was performed using a Procise 494 analyzer (Thermo Scientific).

## Additional Information

**How to cite this article:** Cid, E. *et al*. Non-AUG start codons responsible for *ABO* weak blood group alleles on initiation mutant backgrounds. *Sci. Rep.* **7**, 41720; doi: 10.1038/srep41720 (2017).

**Publisher's note:** Springer Nature remains neutral with regard to jurisdictional claims in published maps and institutional affiliations.

## Supplementary Material

Supplemental Information
