# Supplementary material for: Non-AUG start codons responsible for ABO weak blood group alleles on initiation mutant backgrounds
Source: Sci Rep. 2017 Jan 31;7:41720. doi: 10.1038/srep41720 (PMC5282485; doi:10.1038/srep41720)
Supplement: Supplemental Information [file srep41720-s1.pdf]

Title:

Non-AUG start codons responsible for *ABO* weak blood group alleles on initiation mutant backgrounds

Authors:

Emili Cid, Miyako Yamamoto, and Fumiichiro Yamamoto

Supplemental Information List:

Supplementary Methods (SM1)

Supplementary Figures (SF1 and SF2)

Supplementary Data (SD1): N-terminal protein sequencing

Supplementary Methods SM1

Oligonucleotide primers used for DNA constructs

N-terminal deletion mutants

Forward:

A:	IM261(hAT_RI_Met1F)	CCCGAATTCGCCATGGCCGAGGTGTTGCGG
Δ19:	IM262(hAT_RI_Met2F)	CCCGAATTCGCCATGATCCTTTTCCTAATA
Δ25:	IM263(hAT_RI_Met3F)	CCCGAATTCGCCATGCTTGTCTTGGTCTTG
Δ42:	IM264(hAT_RI_Met4F)	CCCGAATTCGCCATGCCAGGAAGCCTGGAA
Δ52:	IM265(hAT_RI_Met5F)	CCCGAATTCGCCATGGCTGTTAGGGAACCT
Δ68:	IM266(hAT_RI_Met6F)	CCCGAATTCGCCATGGTCTACCCCCAGCCA
Δ141:	IM267(hAT_RI_Met7F)	CCCGAATTCGCCATGGTGGGCCACCGTGTC
Δ95-W96M:	EC129	AAGAATTCACCATGGAGGGCACATTCAACATCG
Δ112-N113M:	EC131	AAGAATTCACCATGACCACCATTGGGTTAACTGTG
Δ121-I122M:	EC133	TAGAATTCACCATGAAGAAATACGTGGCTTTCCTTG

Reverse:

E822 (HUMANBTBamR) CCGGGATCCTCACGGGTTCCGGACCGCCTGG

myc-tagged versions

T7: TAATACGACTCACTATAGGG

EC84:

TTAGGATCCTCACAGATCCTCTTCTGAGATGAGTTTTTGTTCCGGGTTCCGGACCGCCTGGTGG

Other oligos

EC99:	AATTCTCGAGACCATGAGCAGACTGCTGGG
EC110:	TATGAATTCTCACGGGTTCCGGACCGCCTGG
EC111:	AAAGCTAGCGCCACAACCATGGTGAGCAA
EC112:	TTTTCTCGAGTTACTTGTACAGCTCGTCCATGC

B4GALT2

EC19:	CAGAATTCGGATGAGCAGACTGCTGGGG
EC20:	GCTGACACTAATGGACAGAGGCTGAATTCTG

FUT2

TF49 (F):	GGCATGAGAAGCACATGGTTCCATCTCGAGCGG
TF48 (R):	CCGGAATTCCATGCTGGTCGTTCAGATGCCTTT
TF50 (NESTED R):	CCGCTCGAGTTGAGGGAGGCAGAGAAGGAGAAA

pMigR2g

IM927:	CCGGAATTCCGGATCCCCCCCCTAACGTTA
IM928:	CTTGCTCACCATGGTTGTGGCCATATTATC

Threonine mutants

A-M1,20,26T
N-terminal PCR:
Template pSG5-A cut with *XbaI*

IM667:	CCCGGAATTCCACGGCCGAGGTG
IM670:	CGTTATTAGGAAAAGGATCGTAGGTCGAAG

C-terminal PCR:
Template: pSG5-A cut with *XbaI*

IM669:	ACGATCCTTTTCCTAATAACGCTTGTCTTG
IM340:	CCCGGGATCCGCTCACGGGTTCCGGACCGC

A-M1,69T
N-terminal PCR:
Template: pSG5-A cut with *XbaI*
IM667

IM676:	AGACCGTCCTTGGCAACG

C-terminal PCR:
Template: pSG5-A cut with *XbaI*

IM675:	CGTTGCCAAGGACGGTCTACCC

IM340

A-M1,20,26,43,53,69T
N-terminal PCR:
Template pSG5-A-M1,20,26T cut with *XbaI*

IM667:	CCCGGAATTCCACGGCCGAGGTG
IM672:	TCCTGGCGTTAGACTTCTGG

Central PCR:
Template pSG5-A cut with *XbaI*

IM671:	AAGCCCCAGAAGTCTAACGCCAGGAAGC
IM674:	CCCTAACAGCCGTGCAGAACCC

C-terminal PCR
Template pSG5-A-M1,69T cut with *XbaI*

IM673:	TCTGCACGGCTGTTAGGG

IM340

A-STOP- M1,20,26,43,53,69T
Template: pSG5-A-M1,20,26,43,53,69T cut with *XbaI*

IM750:	CCGGAATTCCTGAGCCACGGCCGAGGTGTTGCGGA

IM340

Supplementary Figures (SFs)

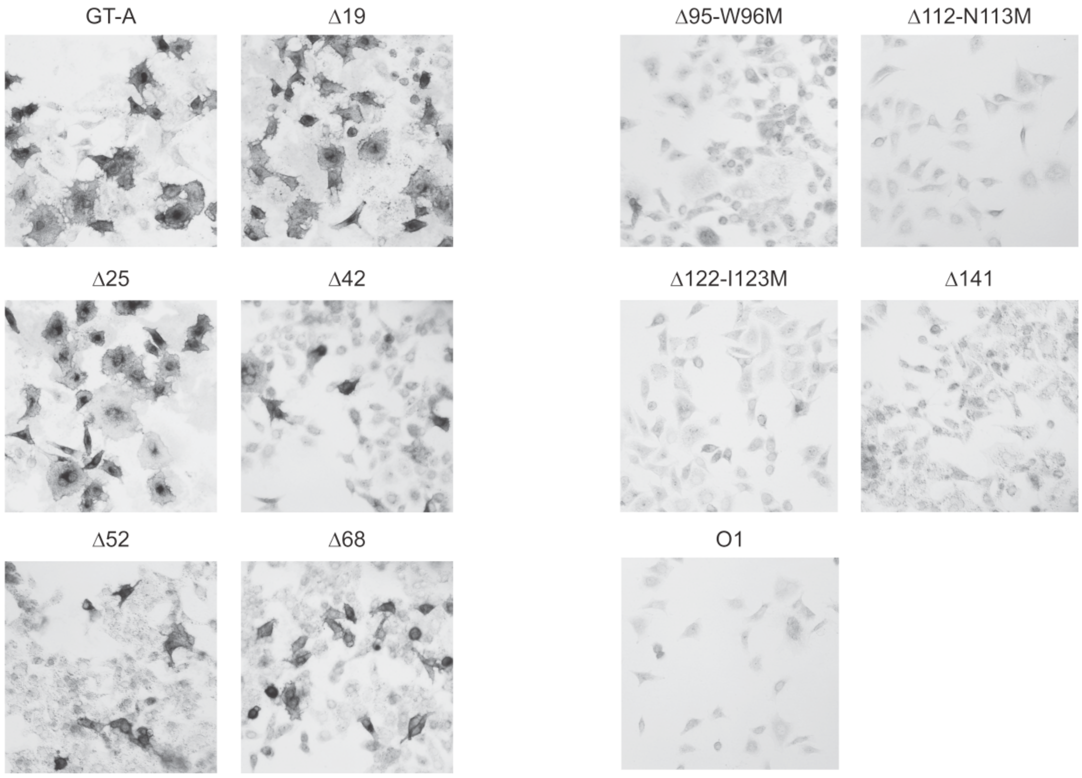

Supplementary Figure SF1: Immunocytochemistry of HeLa FUT2 cells transfected with N-terminal deletion A constructs

Together with pSUPER-Venus, truncated A expressing constructs cloned in pSG5 were transfected in HeLa FUT2 cells using Lipofectamine 2000 reagent in OPTI-MEM medium (both from Life Technologies). Cells were plated in 96 well plates and DNA transfection experiments were performed following the protocol provided by the manufacturer. Transfection medium was replaced 15 h after transfection. Three days after, cells were fixed with 4% paraformaldehyde and washed extensively with PBS. For immunocytochemistry, cells were blocked with diluted goat serum for 30 min. Anti-A antibody (Ortho Clinical Diagnostics) was used without dilution. Incubation was done at 4°C for 1 h. Cells were washed, and secondary antibody, biotin-conjugated anti-mouse IgM (Vector Laboratories), was applied. After 1 h at 4°C, cells were washed and then incubated at room temperature for 1 h with avidin-biotin immunoperoxidase reagent (Vectastain ABC kit, Vector Laboratories). Then cells were washed with PBS and also with molecular grade water. Staining was performed, by applying fresh diaminobenzidine (DAB) solution (Vector Laboratories) in water. The reaction was allowed to develop for 30–45 min at room temperature. DAB was removed and cells were washed with water and PBS. Images were taken using a Leica DMI 6000B microscope, a 20x objective and a DFC360FX camera and analyzed with Leica LAS software.

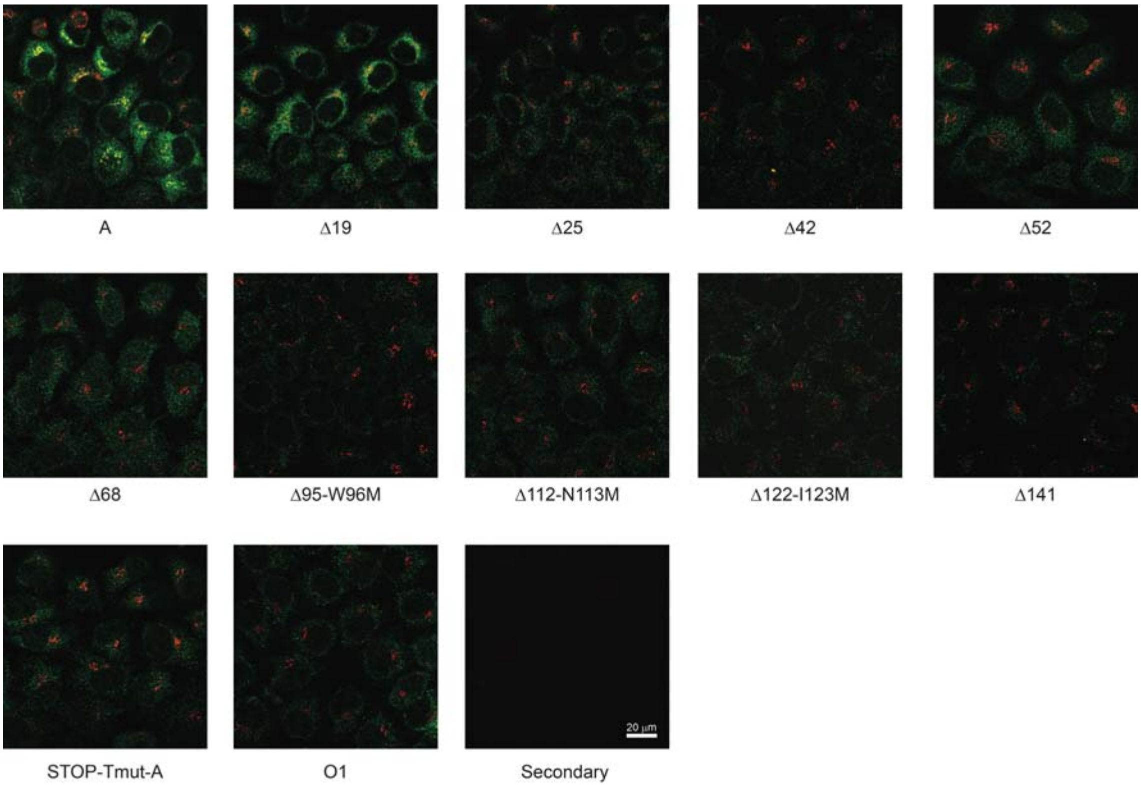

Suplementary Figure SF2: Immunofluorescence of HeLa FUT2 cells stably transfected with myc-tagged N-terminal deletion A constructs

HeLa FUT2 cells were stably transfected with A transferase mutants by retroviral infection and FACS selection. Cells grown in glass coverslips were fixed with 4% paraformaldehyde and subjected to immunostaining with a primary anti-myc antibody followed by secondary Alexa568-anti mouse IgG antibody. GOLGA2, a Golgi protein, was detected using a rabbit anti-GOLGA2 antibody followed by secondary Alexa647-anti rabbit IgG antibody. The merged images are colour-coded green for myc-tagged proteins and red for GOLGA2, showing colocalization in yellow. The “secondary” panel shows the signal of A-myc expressing cells treated with the secondary antibodies in the absence of the primaries. Representative confocal images are shown.

Supplementary Data SD1: N-terminal protein sequencing

The results of the N-terminal sequencing of A-myc and A-STOP-M1,20,26,43,53,69T proteins are appended. In the case of the original A transferase the fourth amino acid could not be assigned due to a technical error. No other AEV sequence is found in the protein sequence.

QUIMICA DE PROTEINAS (CIB-CSIC)
Ramiro de Maeztu, 9
28040 Madrid

PNA-SQP004-F02

SAMPLE INFORMATION

Sample Name:	Emili Cid_A myc	Std Amount:	8.000 pmols
ID Code:		Sample Amount:	0.000 pmols
		Detector Scale:	0.005 AUFS
Comments:	AEVX		

SEQUENCER INFORMATION

Name:	PROCISE	Model Number:	494
Method:	PL PVDF mod	Cartridge:	A
Operator:	Javier Varela		

Cycle 1:Blank 1

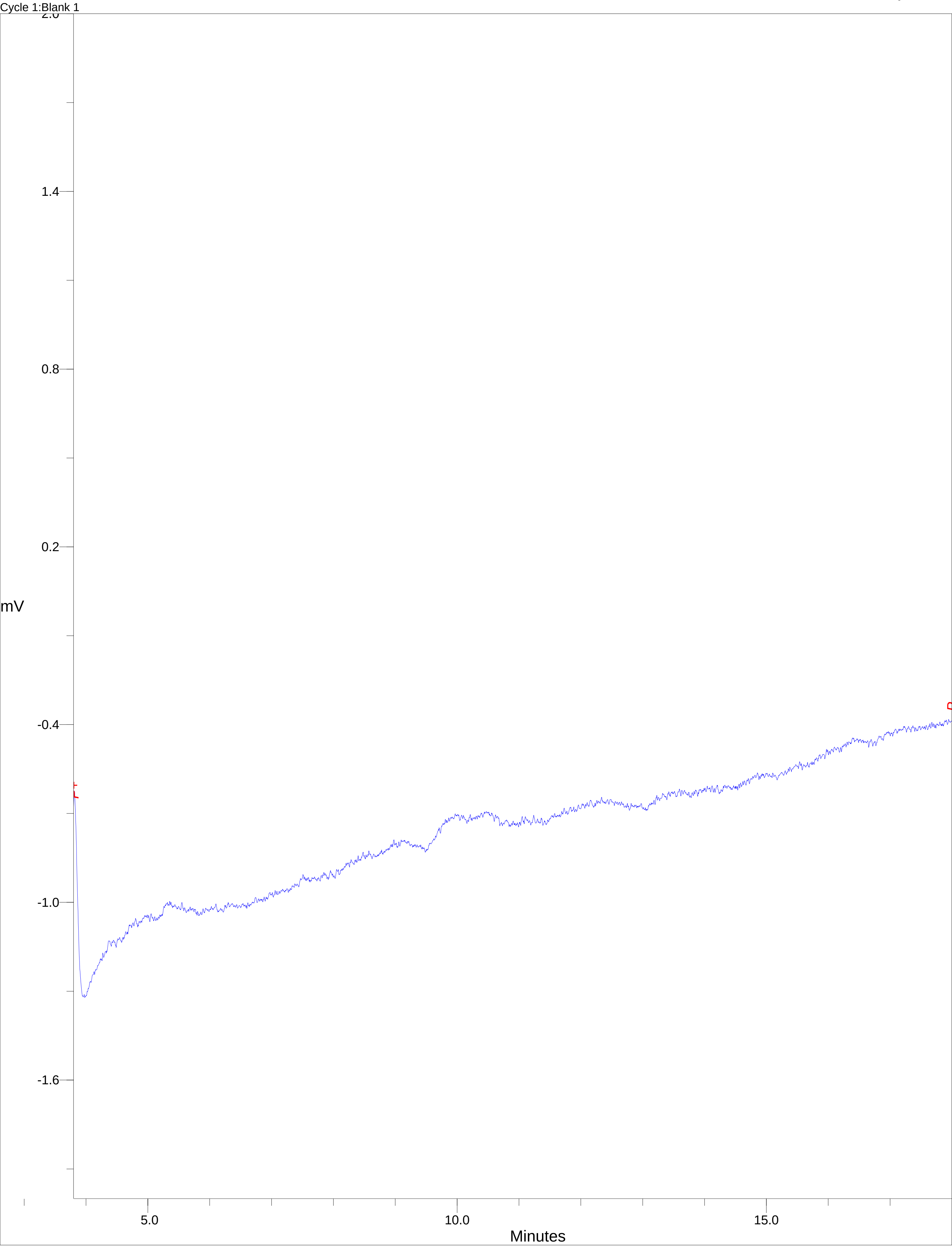

QUIMICA DE PROTEINAS (CIB-CSIC)
Ramiro de Maeztu, 9
28040 Madrid

Cycle 2:Standard 1

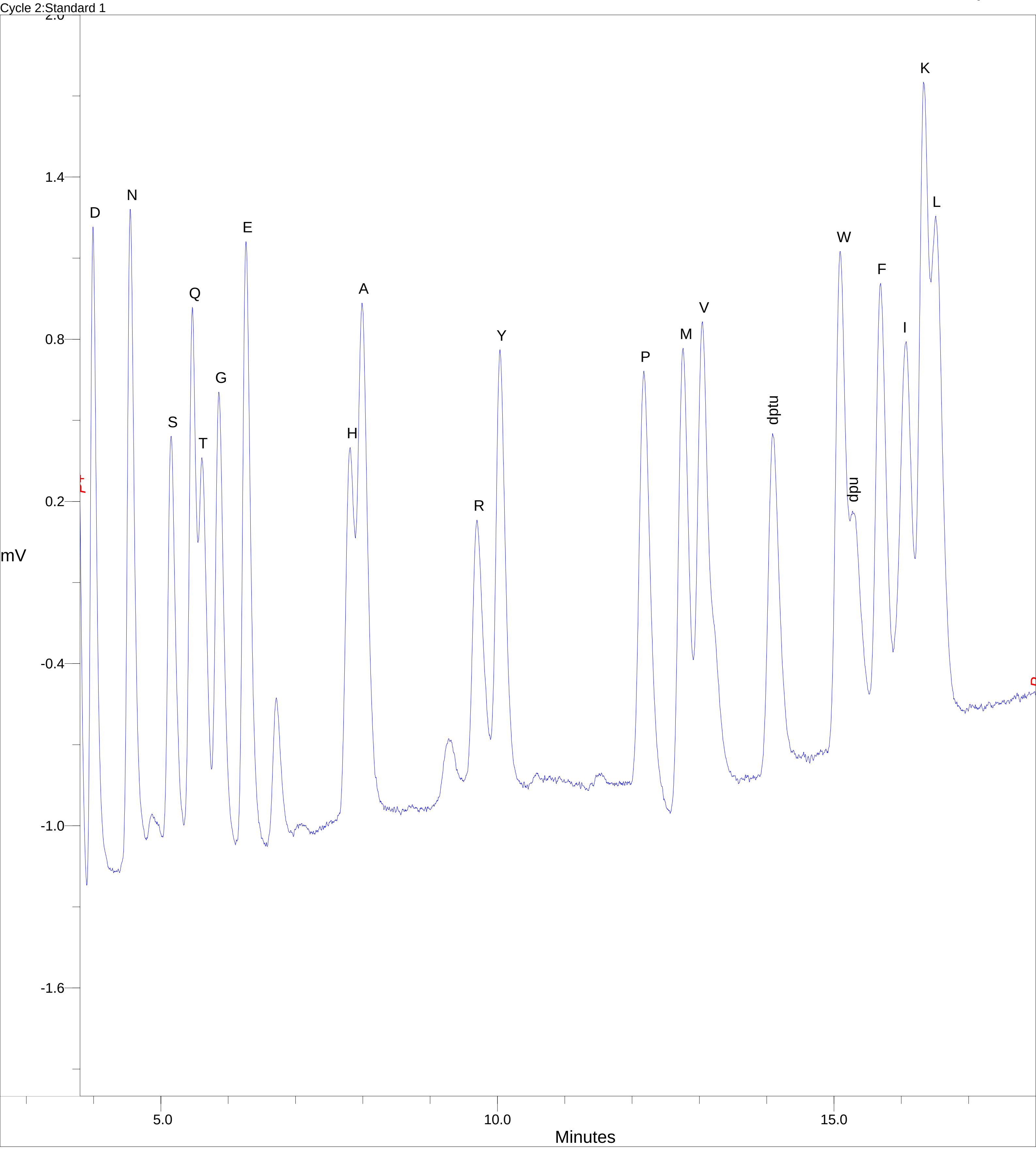

PEAK ID	R.TIME (mins)	C.TIME (mins)	HEIGHT (mV)	PMOL HT
D	3.99	3.99	2.433	8.000
N	4.54	4.54	2.485	8.000
S	5.15	5.15	1.624	8.000
Q	5.47	5.47	2.091	8.000
T	5.61	5.61	1.530	8.000
G	5.86	5.86	1.767	8.000
E	6.26	6.26	2.310	8.000
H	7.81	7.81	1.500	8.000
A	7.99	7.99	2.029	8.000
R	9.70	9.70	1.174	8.000
Y	10.04	10.04	1.794	8.000

PEAK ID	R.TIME (mins)	C.TIME (mins)	HEIGHT (mV)	PMOL HT
P	12.18	12.18	1.649	8.000
M	12.76	12.76	1.707	8.000
V	13.05	13.05	1.782	8.000
dptu	14.09	14.09	1.285	8.000
W	15.09	15.09	1.879	8.000
dpu	15.28	15.28	0.905	8.000
F	15.69	15.69	1.714	8.000
I	16.07	16.07	1.468	8.000
K	16.33	16.33	2.409	8.000
L	16.51	16.51	1.899	8.000

Cycle 3:Residue 1

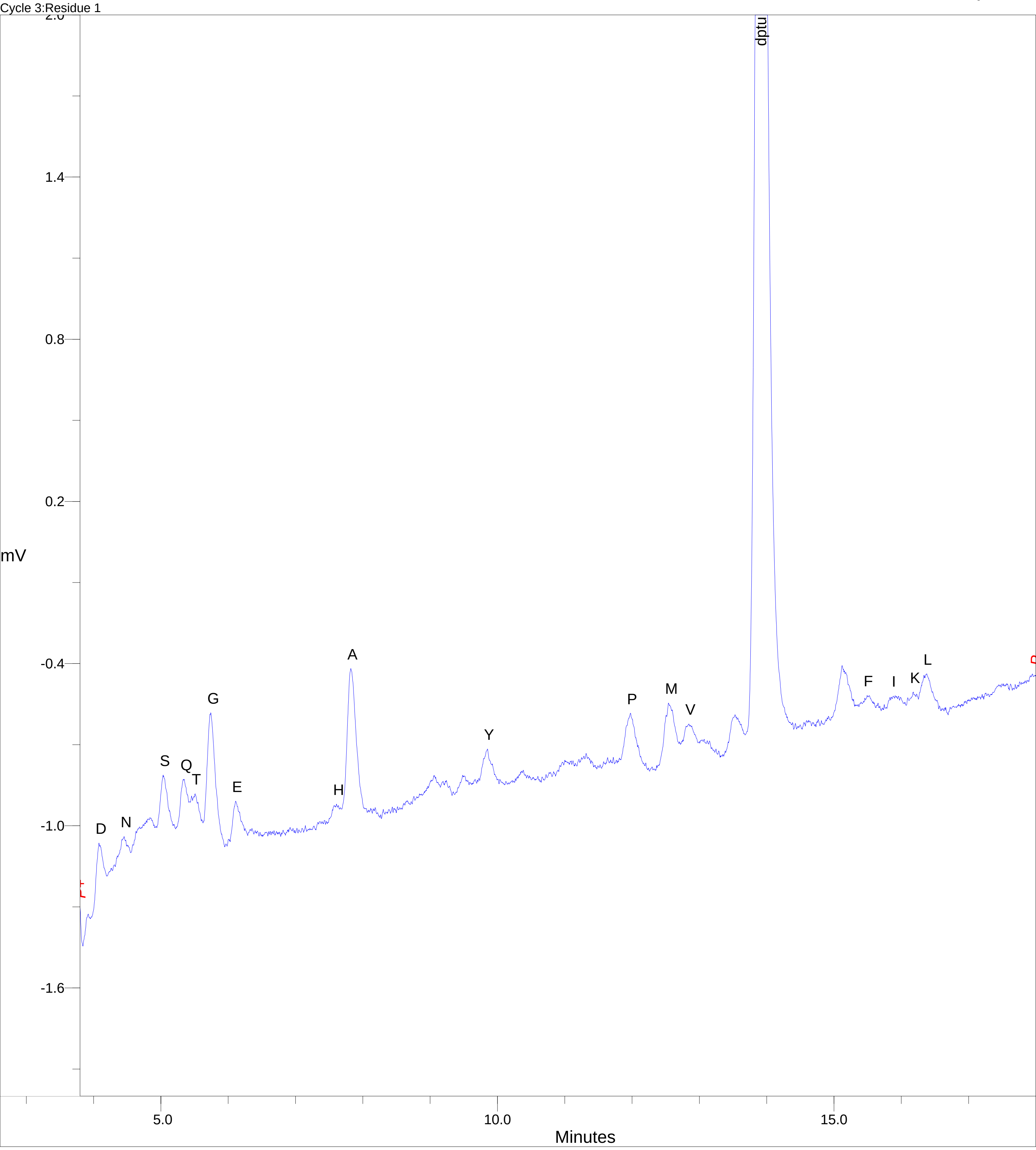

PEAK ID	R.TIME (mins)	C.TIME (mins)	HEIGHT (mV)	PMOL HT
D	4.08	3.99	0.366	1.205
N	4.45	4.54	0.365	1.176
S	5.03	5.15	0.554	2.730
Q	5.34	5.47	0.518	1.982
T	5.51	5.61	0.453	2.371
G	5.74	5.86	0.737	3.340
E	6.11	6.26	0.386	1.338
H	7.61	7.81	0.275	1.468
A	7.82	7.99	0.763	3.007
Y	9.85	10.04	0.330	1.472
P	11.98	12.18	0.321	1.558

PEAK ID	R.TIME (mins)	C.TIME (mins)	HEIGHT (mV)	PMOL HT
M	12.54	12.76	0.321	1.503
V	12.84	13.05	0.224	1.004
dptu	13.91	14.09	5.788	36.023
F	15.49	15.69	0.153	0.715
I	15.91	16.07	0.124	0.677
K	16.18	16.33	0.120	0.399
L	16.38	16.51	0.174	0.734

QUIMICA DE PROTEINAS (CIB-CSIC)
Ramiro de Maeztu, 9
28040 Madrid

Cycle 4:Residue 2

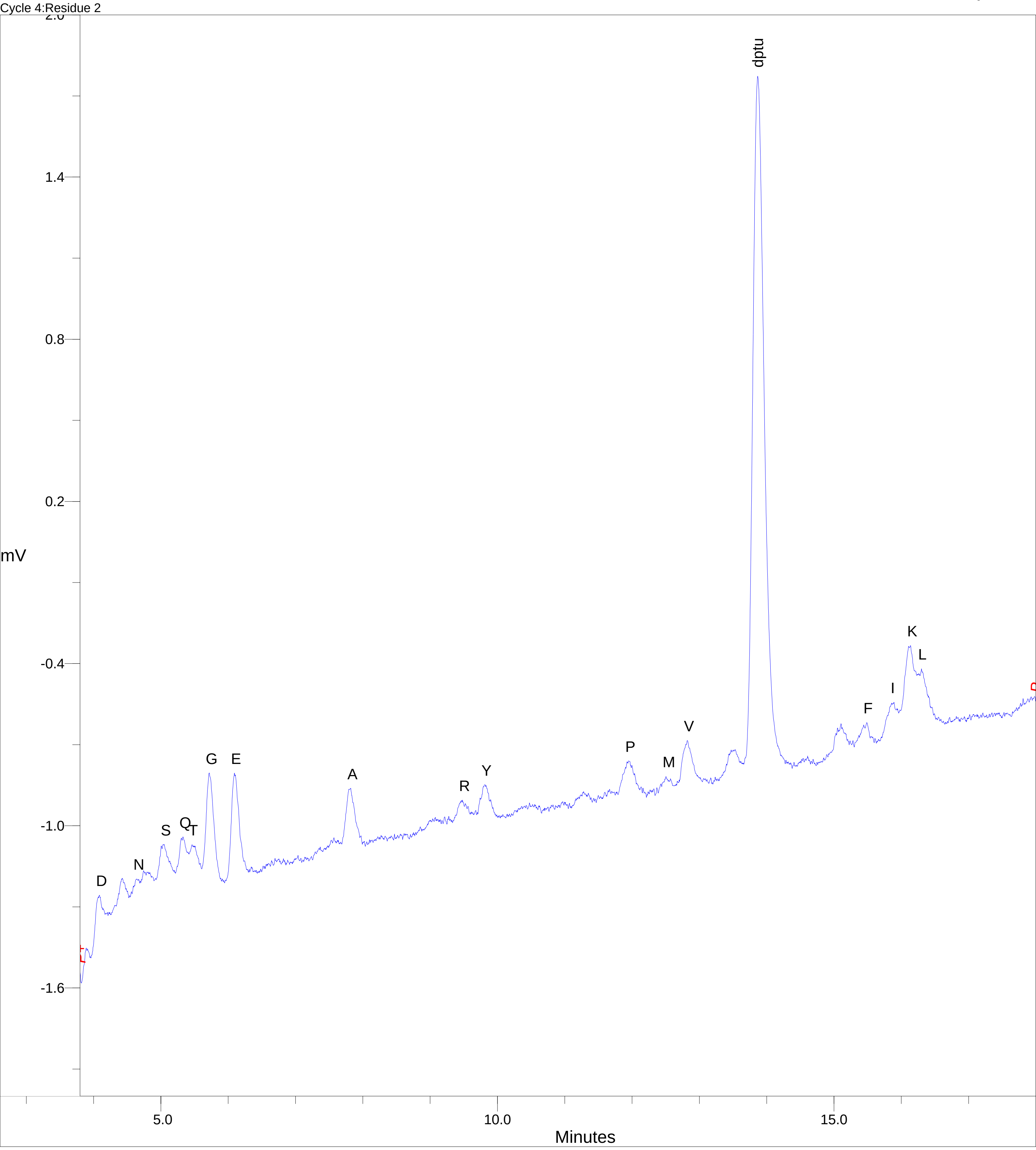

PEAK ID	R.TIME (mins)	C.TIME (mins)	HEIGHT (mV)	PMOL HT
D	4.09	3.99	0.307	1.009
N	4.64	4.54	0.327	1.053
S	5.05	5.15	0.426	2.098
Q	5.33	5.47	0.434	1.661
T	5.46	5.61	0.395	2.066
G	5.72	5.86	0.642	2.908
E	6.09	6.26	0.615	2.131
A	7.82	7.99	0.437	1.722
R	9.48	9.70	0.274	1.865
Y	9.81	10.04	0.306	1.366
P	11.95	12.18	0.242	1.174

PEAK ID	R.TIME (mins)	C.TIME (mins)	HEIGHT (mV)	PMOL HT
M	12.50	12.76	0.145	0.681
V	12.82	13.05	0.255	1.147
dptu	13.87	14.09	2.638	16.421
F	15.49	15.69	0.131	0.614
I	15.89	16.07	0.178	0.968
K	16.14	16.33	0.370	1.230
L	16.30	16.51	0.273	1.149

QUIMICA DE PROTEINAS (CIB-CSIC)
Ramiro de Maeztu, 9
28040 Madrid

Cycle 5:Residue 3

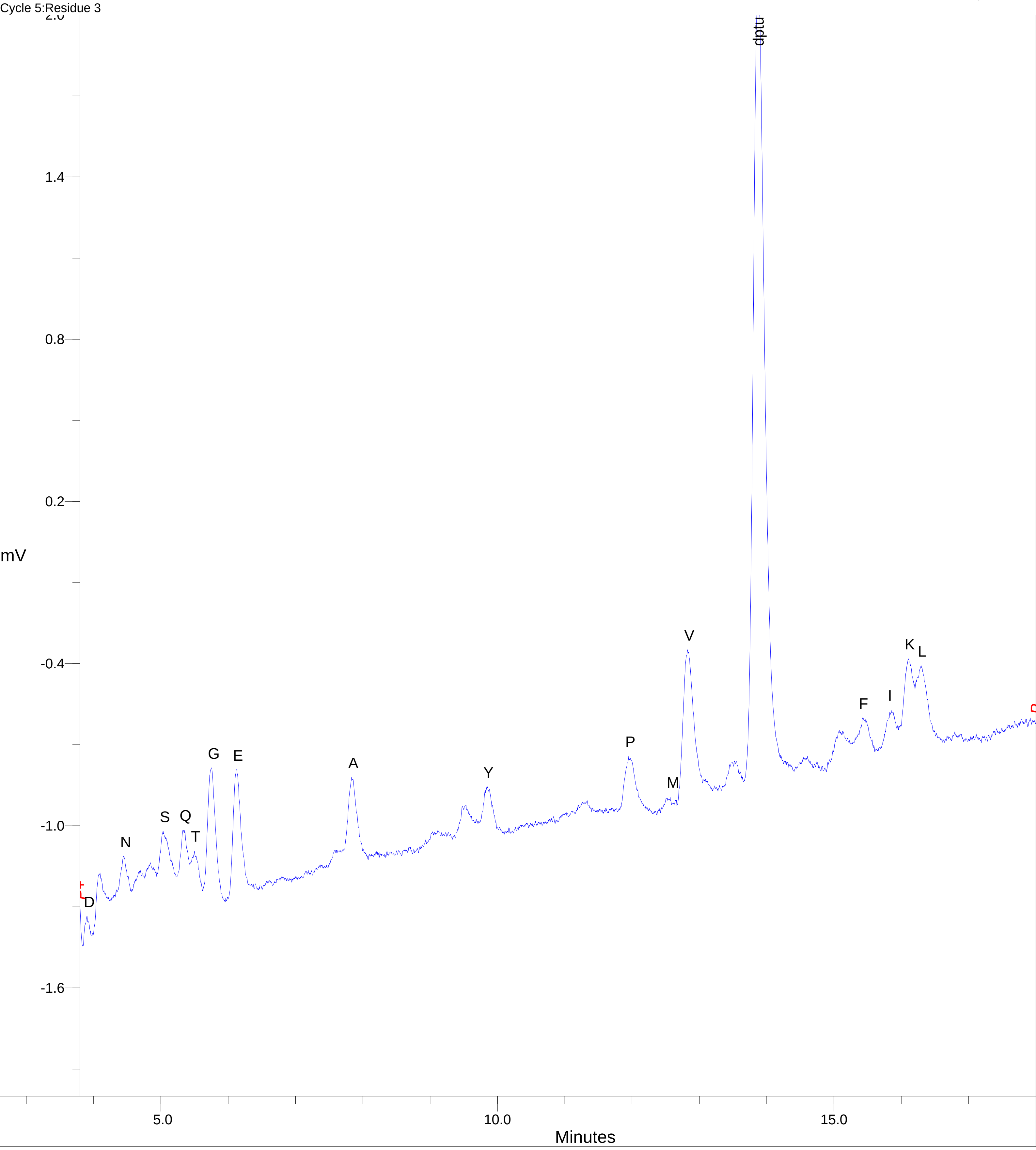

PEAK ID	R.TIME (mins)	C.TIME (mins)	HEIGHT (mV)	PMOL HT
D	3.90	3.99	0.109	0.357
N	4.45	4.54	0.298	0.960
S	5.03	5.15	0.359	1.771
Q	5.33	5.47	0.346	1.325
T	5.50	5.61	0.260	1.358
G	5.75	5.86	0.552	2.502
E	6.12	6.26	0.525	1.818
A	7.83	7.99	0.400	1.577
Y	9.84	10.04	0.252	1.125
P	11.95	12.18	0.248	1.202
M	12.57	12.76	0.061	0.284

PEAK ID	R.TIME (mins)	C.TIME (mins)	HEIGHT (mV)	PMOL HT
V	12.82	13.05	0.591	2.652
dptu	13.88	14.09	3.047	18.967
F	15.42	15.69	0.193	0.903
I	15.85	16.07	0.198	1.077
K	16.10	16.33	0.374	1.243
L	16.30	16.51	0.336	1.416

QUIMICA DE PROTEINAS (CIB-CSIC)
Ramiro de Maeztu, 9
28040 Madrid

Cycle 6:Residue 4

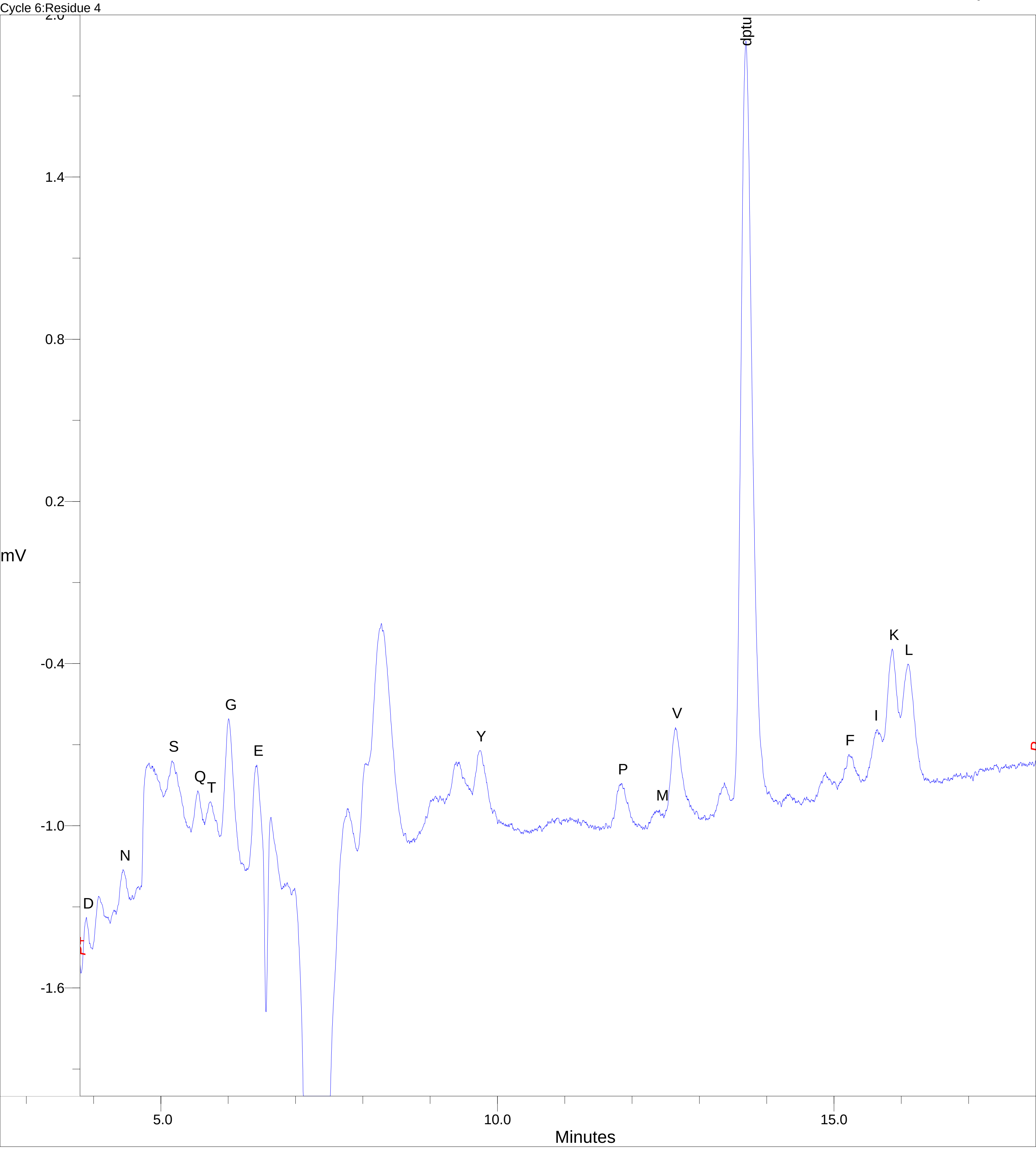

PEAK ID	R.TIME (mins)	C.TIME (mins)	HEIGHT (mV)	PMOL HT
D	3.89	3.99	0.283	0.931
N	4.44	4.54	0.971	3.125
S	5.17	5.15	2.057	10.135
Q	5.55	5.47	2.304	8.812
T	5.73	5.61	2.437	12.744
G	6.00	5.86	3.000	13.582
E	6.42	6.26	3.221	11.155
Y	9.73	10.04	3.135	13.982
P	11.84	12.18	2.227	10.800
M	12.41	12.76	1.918	8.990
V	12.65	13.05	2.134	9.582

PEAK ID	R.TIME (mins)	C.TIME (mins)	HEIGHT (mV)	PMOL HT
dptu	13.69	14.09	4.283	26.656
F	15.22	15.69	1.072	5.001
I	15.65	16.07	1.005	5.477
K	15.87	16.33	1.221	4.056
L	16.10	16.51	1.078	4.543

QUIMICA DE PROTEINAS (CIB-CSIC)
Ramiro de Maeztu, 9
28040 Madrid

PNA-SQP004-F02

SAMPLE INFORMATION

Sample Name:	Emili Cid_M17	Std Amount:	8.000 pmols
ID Code:		Sample Amount:	0.000 pmols
		Detector Scale:	0.005 AUFS
Comments:	AEVL		

SEQUENCER INFORMATION

Name:	PROCISE	Model Number:	494
Method:	PL PVDF mod	Cartridge:	A
Operator:	Javier Varela		

Cycle 1:Blank 1

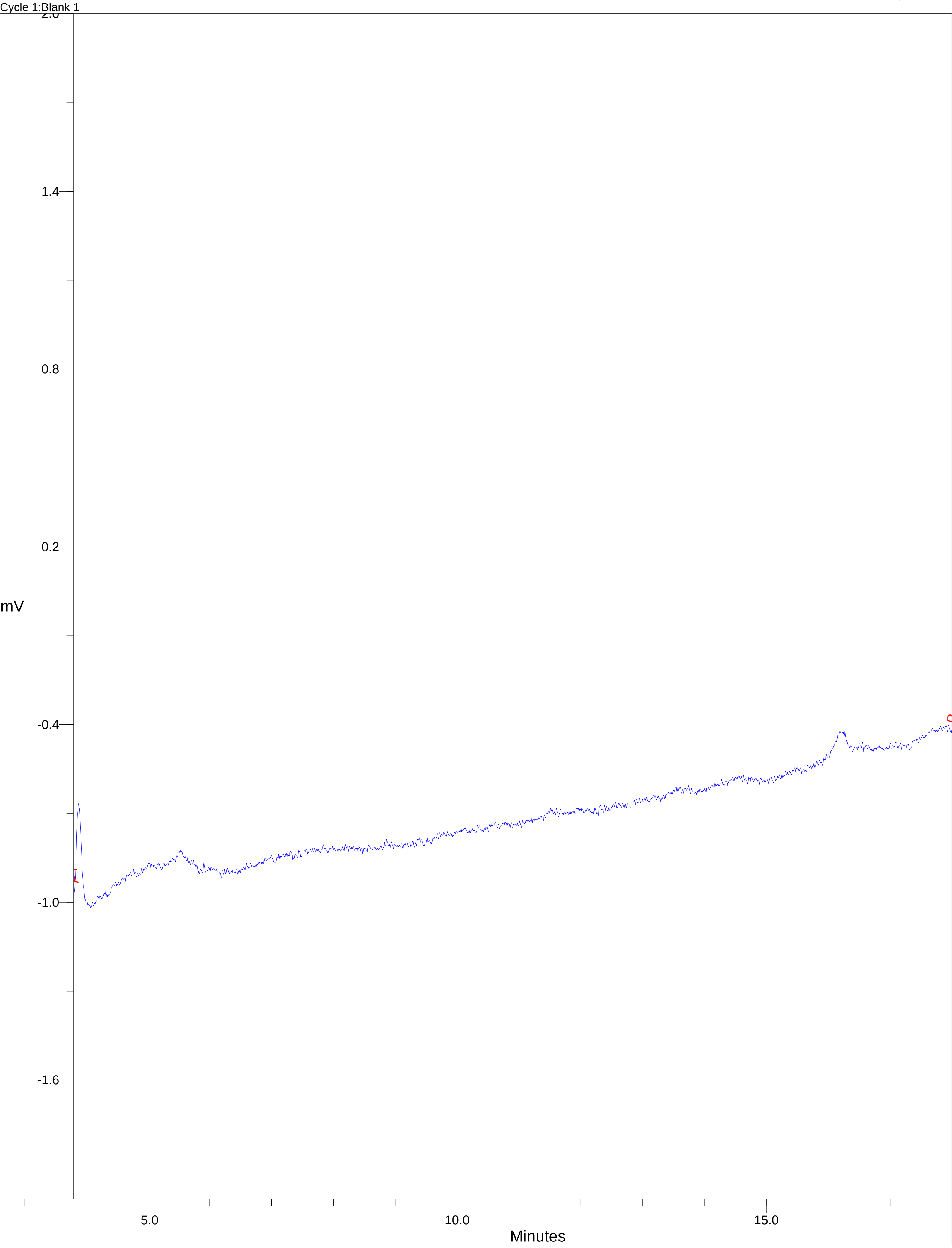

Cycle 2:

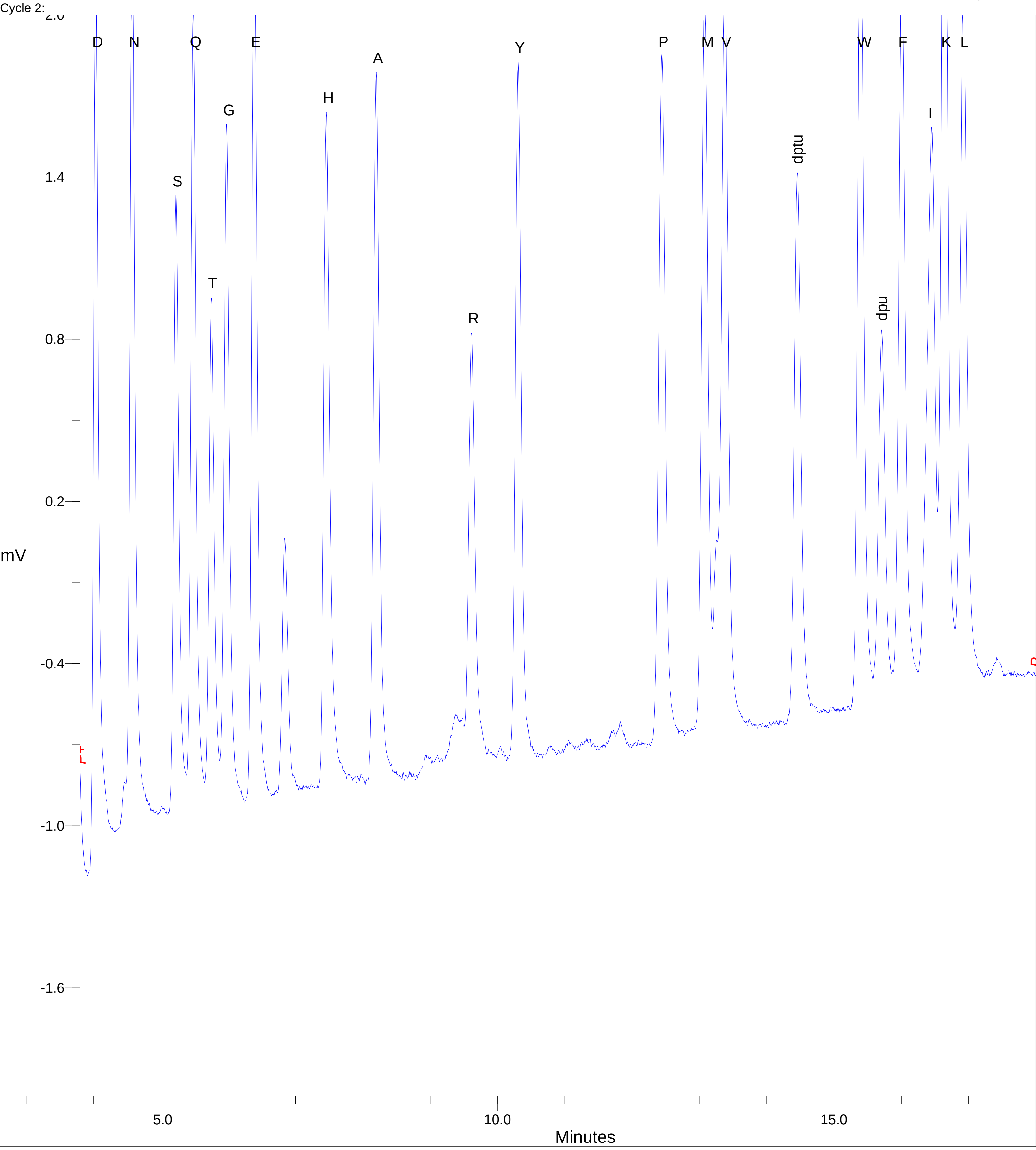

PEAK ID	R.TIME (mins)	C.TIME (mins)	HEIGHT (mV)	PMOL HT
D	4.03	4.03	3.397	8.000
N	4.58	4.58	3.718	8.000
S	5.22	5.22	2.453	8.000
Q	5.48	5.48	3.152	8.000
T	5.75	5.75	2.042	8.000
G	5.97	5.97	2.671	8.000
E	6.39	6.39	3.480	8.000
H	7.46	7.46	2.639	8.000
A	8.20	8.20	2.747	8.000
R	9.61	9.61	1.710	8.000
Y	10.31	10.31	2.674	8.000

PEAK ID	R.TIME (mins)	C.TIME (mins)	HEIGHT (mV)	PMOL HT
P	12.44	12.44	2.591	8.000
M	13.08	13.08	2.788	8.000
V	13.38	13.38	2.893	8.000
dptu	14.45	14.45	2.047	8.000
W	15.40	15.40	3.125	8.000
dpu	15.71	15.71	1.399	8.000
F	16.00	16.00	2.823	8.000
I	16.45	16.45	2.109	8.000
K	16.64	16.64	3.919	8.000
L	16.93	16.93	2.707	8.000

Cycle 3:

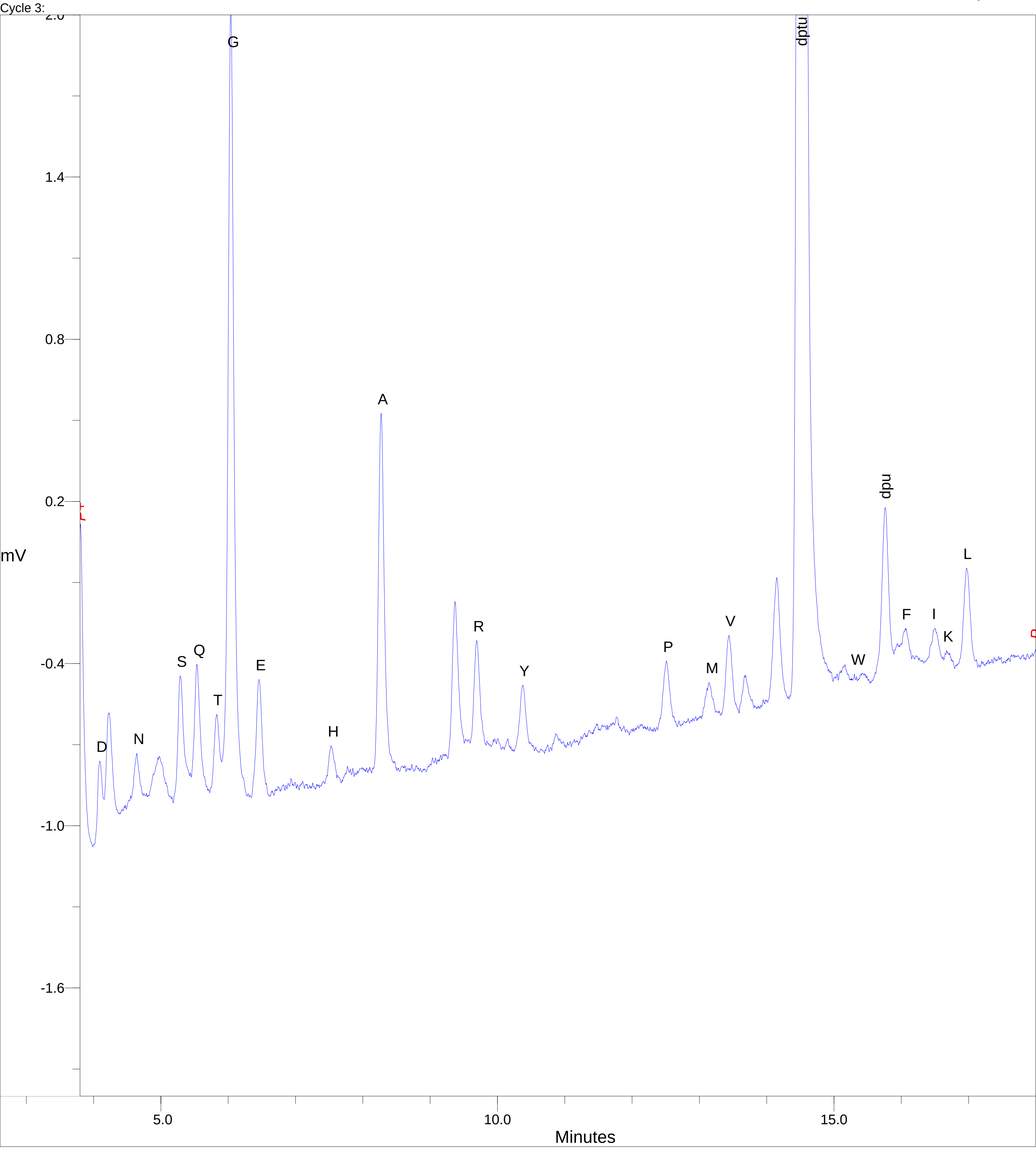

PEAK ID	R.TIME (mins)	C.TIME (mins)	HEIGHT (mV)	PMOL HT
D	4.09	4.03	0.312	0.735
N	4.64	4.58	0.314	0.676
S	5.29	5.22	0.568	1.852
Q	5.53	5.48	0.599	1.519
T	5.83	5.75	0.398	1.560
G	6.04	5.97	3.013	9.023
E	6.46	6.39	0.496	1.139
H	7.53	7.46	0.198	0.600
A	8.27	8.20	1.391	4.051
R	9.69	9.61	0.479	2.241
Y	10.38	10.31	0.279	0.835

PEAK ID	R.TIME (mins)	C.TIME (mins)	HEIGHT (mV)	PMOL HT
P	12.51	12.44	0.262	0.809
M	13.15	13.08	0.151	0.432
V	13.44	13.38	0.310	0.857
dptu	14.52	14.45	16.415	64.142
W	15.30	15.40	0.074	0.190
dpu	15.76	15.71	0.667	3.814
F	16.06	16.00	0.205	0.581
I	16.50	16.45	0.183	0.694
K	16.67	16.64	0.092	0.188
L	16.97	16.93	0.383	1.131

Cycle 4:

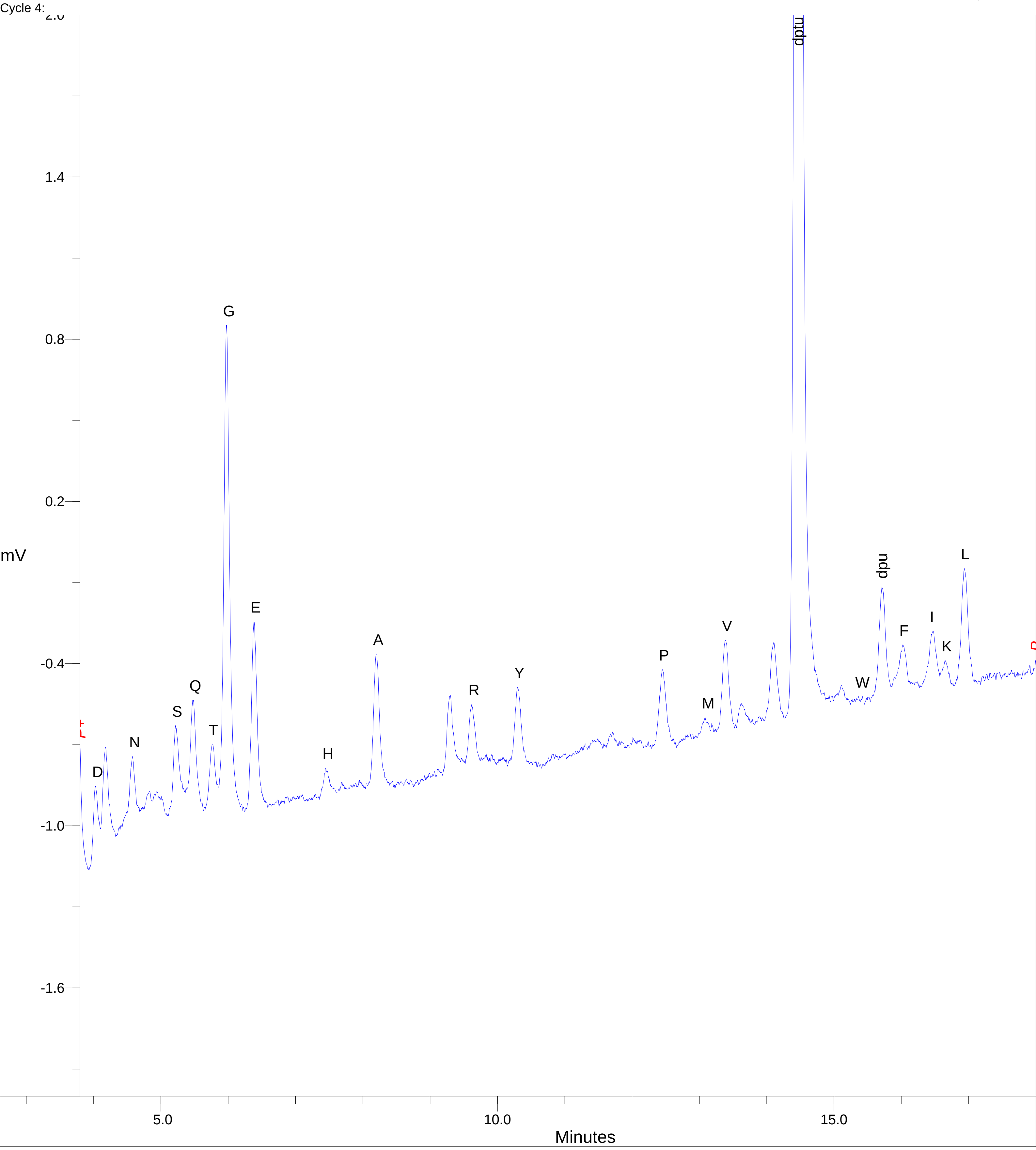

PEAK ID	R.TIME (mins)	C.TIME (mins)	HEIGHT (mV)	PMOL HT	PEAK ID	R.TIME (mins)	C.TIME (mins)	HEIGHT (mV)	PMOL HT
D	4.03	4.03	0.307	0.723	P	12.45	12.44	0.308	0.951
N	4.58	4.58	0.388	0.834	M	13.09	13.08	0.098	0.281
S	5.22	5.22	0.471	1.535	V	13.39	13.38	0.368	1.019
Q	5.47	5.48	0.552	1.400	dptu	14.47	14.45	9.771	38.181
T	5.76	5.75	0.372	1.458	W	15.37	15.40	0.059	0.152
G	5.97	5.97	1.913	5.728	dpu	15.71	15.71	0.446	2.552
E	6.38	6.39	0.794	1.826	F	16.02	16.00	0.218	0.617
H	7.45	7.46	0.199	0.604	I	16.48	16.45	0.246	0.932
A	8.20	8.20	0.582	1.696	K	16.65	16.64	0.127	0.259
R	9.62	9.61	0.324	1.518	L	16.94	16.93	0.453	1.339
Y	10.30	10.31	0.351	1.051					

QUIMICA DE PROTEINAS (CIB-CSIC)
Ramiro de Maeztu, 9
28040 Madrid

Cycle 5:

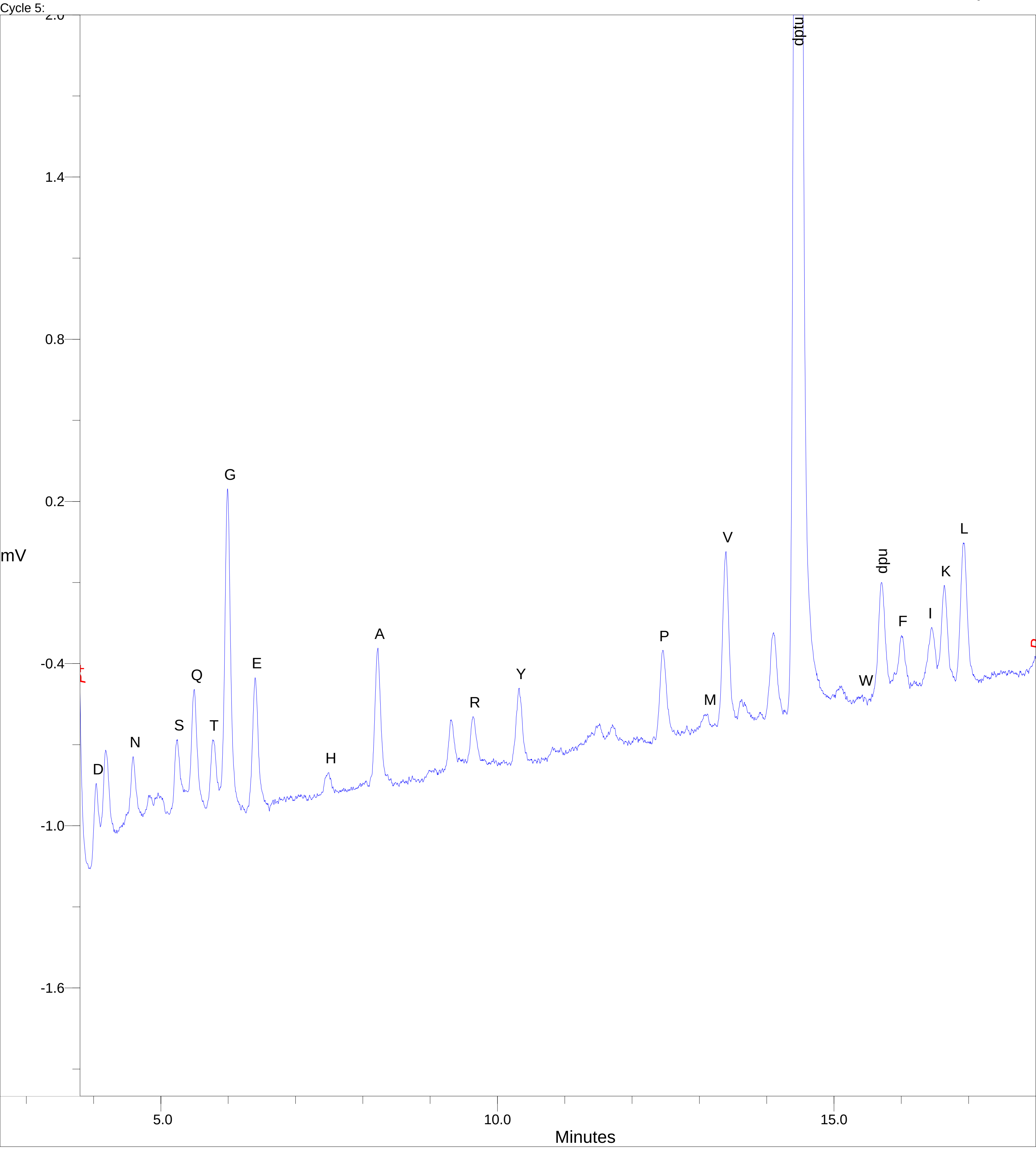

PEAK ID	R.TIME (mins)	C.TIME (mins)	HEIGHT (mV)	PMOL HT
D	4.04	4.03	0.312	0.734
N	4.59	4.58	0.383	0.824
S	5.25	5.22	0.413	1.346
Q	5.50	5.48	0.585	1.485
T	5.77	5.75	0.385	1.508
G	5.99	5.97	1.302	3.900
E	6.40	6.39	0.582	1.338
H	7.50	7.46	0.175	0.532
A	8.22	8.20	0.598	1.743
R	9.63	9.61	0.272	1.272
Y	10.32	10.31	0.343	1.027

PEAK ID	R.TIME (mins)	C.TIME (mins)	HEIGHT (mV)	PMOL HT
P	12.45	12.44	0.372	1.150
M	13.12	13.08	0.102	0.293
V	13.40	13.38	0.690	1.908
dptu	14.46	14.45	9.575	37.417
W	15.42	15.40	0.055	0.141
dpu	15.71	15.71	0.456	2.606
F	16.00	16.00	0.244	0.692
I	16.45	16.45	0.251	0.953
K	16.64	16.64	0.398	0.812
L	16.92	16.93	0.541	1.598

QUIMICA DE PROTEINAS (CIB-CSIC)
Ramiro de Maeztu, 9
28040 Madrid

Cycle 6:

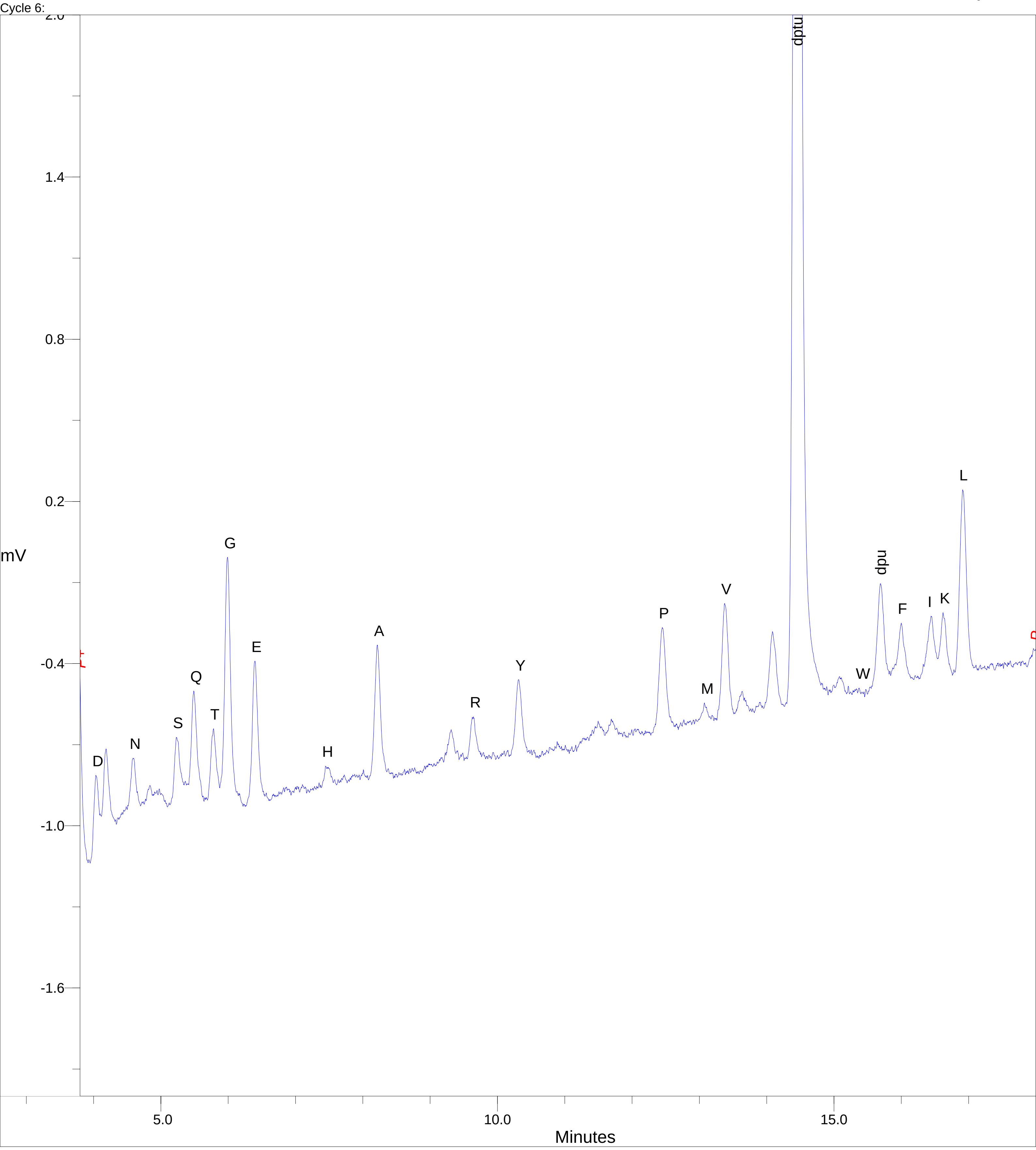

PEAK ID	R.TIME (mins)	C.TIME (mins)	HEIGHT (mV)	PMOL HT
D	4.03	4.03	0.322	0.758
N	4.59	4.58	0.356	0.766
S	5.23	5.22	0.399	1.303
Q	5.49	5.48	0.558	1.417
T	5.78	5.75	0.403	1.580
G	5.99	5.97	1.027	3.076
E	6.40	6.39	0.621	1.427
H	7.45	7.46	0.178	0.539
A	8.22	8.20	0.586	1.706
R	9.64	9.61	0.245	1.148
Y	10.32	10.31	0.348	1.040

PEAK ID	R.TIME (mins)	C.TIME (mins)	HEIGHT (mV)	PMOL HT
P	12.45	12.44	0.429	1.325
M	13.07	13.08	0.118	0.338
V	13.38	13.38	0.470	1.299
dptu	14.45	14.45	8.867	34.648
W	15.38	15.40	0.053	0.135
dpu	15.69	15.71	0.423	2.418
F	16.00	16.00	0.260	0.738
I	16.44	16.45	0.263	0.999
K	16.62	16.64	0.267	0.545
L	16.91	16.93	0.706	2.088
